# Source of Raw Materials and Its Processing for the Manufacturing of Ptolemaic Faience Bowls from Tell Atrib (Nile Delta, Egypt)

**DOI:** 10.3390/ma15186251

**Published:** 2022-09-08

**Authors:** Jerzy Trzciński, Małgorzata Zaremba, Krzysztof Nejbert, Grzegorz Kaproń

**Affiliations:** 1 Institute of Archaeology, Cardinal Stefan Wyszynski University, Wóycickiego 1/3, 01-938 Warsaw, Poland; 2 Faculty of Geology, University of Warsaw, Żwirki i Wigury 93, 02-089 Warsaw, Poland

**Keywords:** faience body and glaze, accessory grains, ore minerals, grain size and shape, crushing and grinding, provenance of quartz

## Abstract

The provenance of siliceous grain material, the basic source of manufacturing faience items, is still a matter of discussion. The study methods applied so far have not brought satisfactory outcomes, and the results are ambiguous and problematic. Archaeological evidence has also not supplied adequate proof for establishing the sites where the source material was obtained and the methods of its preparation. Therefore, we propose an interdisciplinary approach to solve these research problems. We explore selected material of 7 faience bowls precisely dated on the c. 100 years of the Ptolemaic Period in Egypt. The body and glaze of the faience bowls was qualitatively and quantitatively tested with regard to chemical and mineral composition, and selected material parameters. Based on structural-textural analysis, as well as chemical and mineral composition, the source area of the studied raw material and its potential excavation site was determined in the Eastern Desert. The obtained results were compared with locations of mines exploiting gold-bearing quartz veins, functioning in the Ptolemaic Period. Material parameters obtained from image analysis have been applied to reconstruct the processes of crushing and grinding of the quartz material and its further treatment for faience manufacturing. Quartz treatment was analysed with regard to tools and handling processes applied in Ptolemaic mines. We assume that such an approach has given accurate results in determining the provenance of siliceous material used in the Ptolemaic workshops of Athribis. Therefore, in material studies of artefacts produced in the antiquity, it is indispensable to use an interdisciplinary and complex approach, beginning from field studies and ending with detailed laboratory analyses.

## 1. Introduction

The basic raw material used for the manufacturing of faience artefacts in ancient Egypt was silica. After crushing and mixing with additives, it was used to prepare the ceramic mass, i.e., the silica paste, out of which the faience body was formed. The same silica material with other additives was used to prepare the glazing powder and then slurry, which was applied on the body surface. After firing, the faience objects attained a white, light grey or light pink core usually covered with a blue or green glaze [1].

The dominating opinion in the literature indicates that the main source of siliceous material was quartz of diverse origin. In the Tell el-Amarna site, Petrie [2,3] analysed beads composed of very fine white grains. He suggested the application of powdered quartz derived from the crushing and grinding of white quartz pebbles, commonly found in the contemporary workshops. Detailed studies of white powder from this site suggest that the source could have been finely ground quartzites, which were used to prepare the silica paste for faience manufacturing [4]. Lucas [5] analysed typical Egyptian faience from the Predynastic period and later times. The body material, whether fine or coarse, when examined microscopically is seen to consist of sharp, angular quartz grains. He suggested that for the white body material only three origins seem possible: powdered quartz rock, powdered rock crystals, or powdered white quartz pebbles. Vandiver [6], as well as Vandiver and Kingery [7], studied faience objects from ancient Egypt and the Middle East from a time span of several thousand years. These authors suggest the usage of crushed quartz or sand, whose source could have been surface ground sandstone lumps. The usage of quartz sand as the source material was evidenced by the smaller engagement of the workshops in the preparation of finely ground material. Sode and Schnell [8], as well as Nenna and Seif el-Din [9,10], based on studies in sites from the New Kingdom and the Greco-Roman period, have shown that quartz pebbles were used for the manufacturing of faience objects. A total of 21 Ptolemaic faience vessels and vessel fragments from the Walters Art Gallery were studied by Mao [11]. She concluded that the body was made of finely ground (20–100 μm) quartz grains. Shortland and Tite [12] analysed fragments of faience vessels from Memphis (Kom Helul), dated at the Ptolemaic and Early Roman periods. They also suggested that the low content of aluminium oxide in the green glaze points to the usage of powdered quartz, whereas the higher content of this substance in the blue glaze indicates the application of quartz sand. According to them, the core of faience vessels was made from the same source material as the glaze. Tite et al., [13] examined faience ware from Abydos, Amarna and Esna spanning a period from the Middle Kingdom through to the 22nd dynasty. They suggested that the faience vessels were made out of crushed quartz pebbles. Nicholson [14] synthesised information about the production of glass and vitreous materials at Amarna and set it in a wider context of glass and faience production during the New Kingdom. He emphasised that the faience is composed of the same material as glass and frit, i.e., silica. The source of silica for these materials could have been crushed quartz pebbles and sands. However, large amounts of such pebbles were not discovered during the excavations at Kom Helul (Memphis, Roman Period) [15]. Analysis of the faience body by Nenna and Seif el-Din [10] has indicated that objects from the Roman Period contain coarser material than those from the Ptolemaic Period, and comprise single sand grains. Studies of Welc [16,17] and Zaremba et al., [18] of the faience relief-decorated hemispherical bowl from Tell Atrib have allowed to elaborate on the source of the siliceous raw material with precisely dated objects from the Ptolemaic Period. According to this multiproxy approach, the material was pure, high-quality quartz, whose source could have been quartz veins from pegmatites in igneous rocks or coarse-grained clastic quartz rocks. The authors suggest that the material could be derived from the Nile Delta and from the Western Desert, where accumulations of quartz gravels and stones occur, whereas vein quartz could be exploited in Sinai or the Eastern Desert. A lack of rounded quartz grains or their fragments excludes the usage of aeolian sand from the desert.

Indicating the provenance of siliceous material and the reconstruction of the technology of its processing is a complex issue, because there is no access to the siliceous raw material or the silica paste, and the fired material had been altered in high temperatures. The firing process masked the primary features of the material and its transformation processes. So far, no reliable archaeological data exist and no analytical methods have been applied, which would unequivocally determine the source of siliceous material in faience objects.

The application of multiproxy studies: structural-textural, petrographic-mineralogical, geochemical, and image analysis, was focused on determining the type of siliceous rocks that were used as the material for faience manufacturing. The obtained results allowed for the determination of the origin of the quartz, potential mining sites and reconstruction of the methods of its processing for the preparation of the silica paste.

## 2. Materials and Methods

### 2.1. Archaeological Site

The Tell Atrib site (Greek Athribis) is located in the peripheries of the Benha settlement, about 50 km to the north of Cairo in the central part of the Nile Delta. The ancient city developed on the east bank of the Damietta Branch of the Nile River already in the Old Kingdom [19]. The first long-term archaeological excavations in Tell Atrib were commenced by Kazimierz Michałowski in 1957. They were concentrated around Kom A, which at present is situated within the New Benha district. During the 10-year excavations, the remains of two temples, Taharqa and Amazis, from the Late Period, a complex of Roman baths from the Julio-Claudian Period, and a set of several tens of kilns—limekilns from the Roman and Arabic periods have been discovered [20,21,22,23,24,25]. Moreover, the exposed layers yielded many fragments of artefacts, e.g., faience pieces from the Ptolemaic Period, including relief decorated bowls. In 1969 and 1979–1984, the excavations were conducted by Ruszczyc [26] in the frame of the Polish-Coptic Mission. At that time, fragments of constructions belonging to a bath complex, kilns—limekilns and remains of sacral constructions were recognised.

In 1985, investigations were initiated to the south of Kom Sidi Yousuf and on farmlands surrounding the Kom from the north, where geophysical surveys and soundings were performed [27]. In some locations, undisturbed layers from the Ptolemaic and Roman-Byzantine periods were determined [28]. With regard to topography, two architectural complexes differing in structure and function were distinguished. One of them included workshops [28,29]. Among the many finds, a large group of artefacts comprised fragments of faience pieces, including bowls with rarely found decorations [16,30]. Owing to numerous coins and stamps on imported amphoras found in the undisturbed layers, the age of the faience objects could be determined precisely [17]. Rich numismatic and ceramic material allowed to conclude on the age of layers which were distinguished in the entire area of the Ptolemaic workshop district. Precise dating often narrowed the age of the layers to the reign of one or two rulers [28,31,32,33]. The stratigraphy of layers in the Ptolemaic district presented by Myśliwiec [28,31] is as follows:

(a) From the beginning of the Ptolemaic Period (sometimes also the end of the Late Period) until the first decades of the 2nd century BC,
(b) The reign of Ptolemy VI and the second half of the 2nd century BC,
(c) The end of the Ptolemaic Period (1st century BC) until the beginning of the Roman period (1st century BC).

### 2.2. Faience Objects

**Figure 1:**
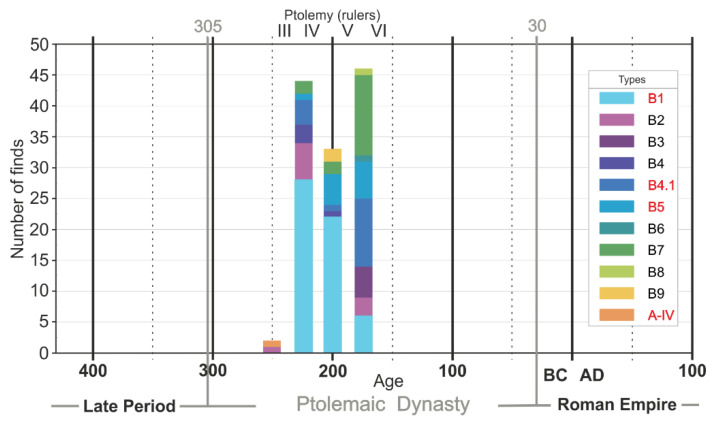
Distribution of the faience bowl types from Tell Atrib according to their dating. Examined types marked in red.

In the Tell Atrib site, 332 fragments of faience objects were found; their typology was presented by Welc [17]. From the entire population of these finds, 285 fragments were located in layers (a) and (b), which allowed for a precise dating. The most frequent were various types of bowls (125 objects), dated at an interval from the middle of the 3rd century BC to the first half of the 2nd century BC (Figure 1). The most numerous is type B.1 (56 finds), while types B.7, B4.1, B.5 and B.2 occur less frequently (10–17 finds). The remaining types are represented by single finds. Moreover, one bowl with a style resembling Achaemenid forms (type A-IV) was also discovered.

**Figure 2:**
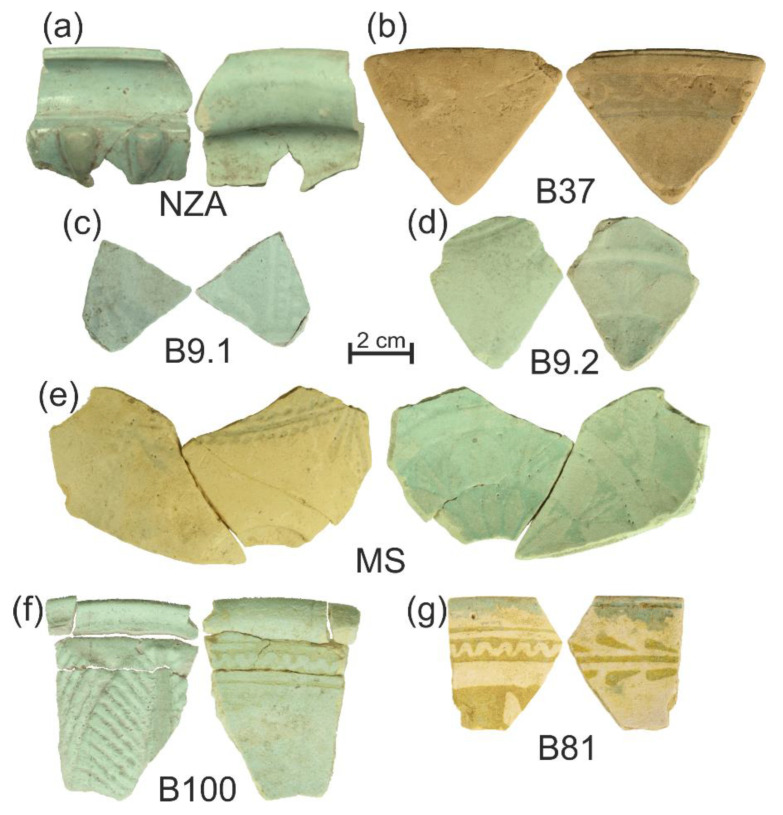
Examined fragments of the faience bowls from Tell Atrib. (**a**–**g**) image pairs with lab numbers: on the left—outside, on the right—inside.

Fragments of 7 bowls, constituting representative material both in terms of typology and age, have been selected for detailed analysis (Table 1, Figure 1). The selected material represents a timespan of about 100 years, during which the largest number of faience bowls was found in the site. The body of selected bowls is white or yellow-white in colour, their thickness varies from 3 to 7 mm, and they are covered by a thin layer of blue or greenish-blue glaze (for detail see Figure 2 and Figure S1 and Table 1).

### 2.3. Preparation of Thin Sections

The samples were cut from fragments of faience vessels to prepare thin sections suitable for microscopic examinations. All thin sections were oriented perpendicular to the surface, parallel to the axis of the vessel, and mounted on a microscopic glass (26 × 48 mm in size). After cutting out, the samples were impregnated with epoxy resin in vacuum conditions. The impregnated samples were glued to the glasses, and then pulverised to the thickness of about 0.025 mm (25 μm). The surface of the thin sections was polished to a smoothness that allowed analysis at large magnifications using the SEM-EDS analytical technique.

### 2.4. Digital and Polarized Light Microscopy

Studies of the faience bowl fragments began from macroscale observations. A Delta Optical Smart 5 MP PRO (Delta Optical, Nowe Osiny, Poland) digital microscope was used for the preliminary analysis. The glaze on the surface and in cross-sections, and the body on the fracture surfaces were analysed.

Analyses of uncoated thin section samples were performed with the application of a petrographic polarization microscope (Nikon Eclipse E600 POL, Nikon Instruments Inc., Melville, NY, USA) coupled with a digital camera (Nikon DS-5Mc) and working with NIS Elements AR software(Ver. 2.30). The studies were focused on textural features of the examined faience, such as identification of primary and secondary minerals, and the textural varieties of the quartz grains used for the manufacturing of the examined faience bowls.

### 2.5. X-ray Powder Diffraction (XRD)

The samples were analysed with an X-ray powder diffractometer X ‘Pert PRO MPD (PANalytical, Malvern Panalytical, Almelo, The Netherlands), using Co Kα radiation (filter Fe) with a PIXcel X-ray detector. The measurements were made using the Bragg-Brentano method in the range 5–78° 2θ, with the step of 0.026° 2θ. The total measurement time of a single sample was up to 10 h. Such a long analysis interval was set to obtain good background statistics of the diffractograms and maximally increase phase traceability.

### 2.6. Scanning Electron Microscopy (SEM) with Energy Dispersive Spectroscopy (EDS)

Thin sections were carbon-coated in a high resolution turbomolecular pumped sputter coater, model Q150T ES produced by Quorum Technologies (Lewes, UK). Carbon-coated thin section samples were observed with Zeiss (Oberkochen, Germany) Σigma™ VP (variable pressure) SEM (scanning electron microscope) with a field emission electron gun (FEG, Oberkochen, Germany) using an AsB™ (Angular selected BSE) detector and EDS analysed using CrossBeam^®^ (Berlin, Germany) Technology EDX (XFlash 6/10™) detectors produced by Bruker. Chamber design provides the ideal geometry for simultaneous two detector EDS analyses. The set conditions were as follows: acceleration voltage 20 kV, beam current 80 µA, working distance 7.7 mm, live time of chemical analysis 60 s. The analysis was executed in high vacuum. Investigations were used to carry out quantitative analysis of the chemical composition. The outstanding hardware is controlled by the ESPRIT software suite. Element identification and spectrum evaluation were made using the standard-less quantification method. This method relies on peak-to-background ZAF evaluation (P/B-ZAF) and provides reliable quantification results.

### 2.7. Petrographic-Mineralogical Analysis

Determination of the provenance of the siliceous material used for the manufacturing of faience was based on the analysis of all mineral and lithic components occurring in the body and glaze, which were jointly referred to as accessory grains with regard to quartz as the dominant component.

The accessory grains occurring in the examined artefacts were used to identify the primary mineralogy of the raw material. The results of the analysis allow for the identification of the genetic type of quartz that was subject to exploitation and are the basis for a discussion on the location of the quartz mining sites. The set of analytical data (SEM and EDS) was used to identify the primary mineral grains, taking into account their changes taking place during the firing process. Identification of minerals was carried out in two stages. In the first step, it was based on the determined chemical composition of the grains, and in the second step, the textures of the tested accessory phases were taken into account. Thermal treatment of the grains during firing may change their mineral composition and, to some extent, affect their texture, while due to the short-term exposure to high temperatures, the original textures were not obliterated completely.

The entire thin section surface was analysed in detail in the SEM-BSE mode at ×100 and ×200 magnifications. The aim of the analysis was to find and identify accessory minerals originally co-existing with quartz in the mineral deposit. Close attention was paid to small inclusions of ore minerals in the quartz grains. Depending on the size of the sample and the presence of mineral phases other than quartz, 150 to 500 EDS analyses were conducted in each sample, along with detailed petrographic and mineral documentation (SEM-BSE images, magnifications from ×500 to ×5000 were used most frequently) of the examined grains. The results of the research were compiled in a consistent database containing all the accessory minerals present in the faience body and glaze. The primary accessory minerals identified in the samples were combined into mineral associations occurring in various types of mineral deposits in which quartz is the main gangue component.

### 2.8. Image and Statistical Analysis

The thin section samples were viewed in a back-scattered electron (BSE) mode after coating with a layer of carbon. Photographs were used to carry out image analysis. Quantitative image analysis was based on BSE images with application of STIMAN software [18,34]. The analysis was performed on one image at ×100 magnification on a total area of 6.6 mm^2^. The application of these methods enabled for a detailed characteristic of the microstructure and microtexture of the grain skeleton. The obtained data were used to solve issues related with the technological processes, especially preparation of raw materials, in the manufacturing of faience items.

As a result of all investigations, four datasets were prepared for statistical analysis: (1) database of the faience bowl fragments according to type and dating, (2) database of SEM-BSE images, (3) database of EDS quantitative analysis of the chemical composition, and (4) database of quantitative microstructural and microtextural parameters. The matrix of the correlation coefficient was obtained from the database of microstructural and microtextural parameters using Microsoft Excel (Microsoft Corporation, Microsoft Office Professional plus 2016, Redmond, WA, USA). The most significant and most representative correlation relationships were selected from this matrix.

## 3. Results

### 3.1. X-ray Powder Diffraction (XRD) Analysis of the Body Layer

Quartz is the dominating phase in the samples (Figure S2a). Small calcite contents (B37, B9.1, B9.2, MS) as well as small cristobalite contents (B9.2 and B100) have been detected in some cases (Figure S2b). The presence of cristobalite can be linked to the temperature in which the faience was fired. In each of the diffractograms, there were very weak singular reflections, which could not be reliably connected to specific phases. With all certainty it can be asserted, however, that aside from quartz, other crystalline components of the samples constitute a very small share of maximally a few percent of the sample total content. The small calcite content recognised in a few samples is secondary and derives from the Nile Delta environment, in which the fragments of faience vessels were found during the excavations [35]. Its presence in the samples does not affect the obtained results.

Despite the application of a very long registration time span, the obtained results have not allowed for the identification of other phases in the faience body. Minerals other than quartz occur in the faience in very small amounts and are randomly distributed, which hampers their identification due to the strong background from quartz. The influence of random sample selection for analysis is visible in the comparison of results obtained for sample MS presented by Zaremba et al., [18], in which feldspars and plagioclases were identified beside quartz and cristobalite at short registration time. The application of different analysis variants in the case of samples of the faience body have not given reliable results and is not recommended. In turn, identification of phases which allow to determine the firing temperature may give consistent outcomes.

### 3.2. Petrographic-Mineralogical Analysis of the Grains

#### 3.2.1. Quartz Grains in the Body Layer

**Figure 3:**
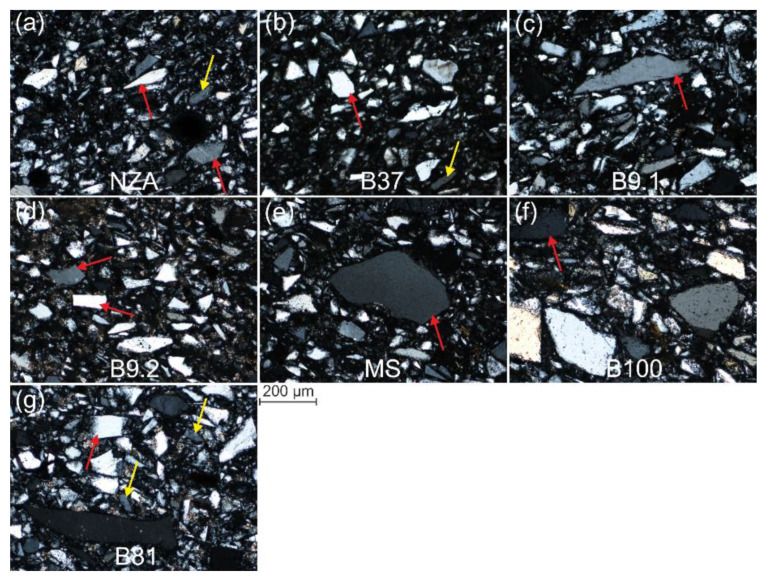
Microstructure of the faience body layer observed under a polarized light microscope. (**a**–**g**) microphotographs with lab numbers. Images in cross-polarized light, 2 nicols, magnification ×100. Yellow arrows indicate feldspars grains, red arrows indicate quartz grains.

**Figure 4:**
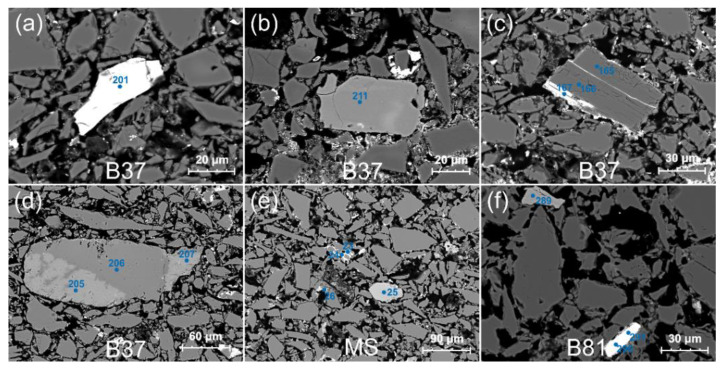
Accessory grains of the faience body and glaze layer observed in a scanning electron microscope (SEM); category A—accessory minerals with an unchanged texture. (**a**–**f**) microphotographs with lab numbers viewed in the back-scattered electron mode (BSE). Magnification: (**a**) ×3000, (**b**) ×2500, (**c**) ×2000, (**d**) ×1000, (**e**) ×700, (**f**) ×2000. Location of EDS analyses marked with blue numbers and dots (see Tables S1 and S2 for elemental and mineral composition).

Quartz is the main component of the body layer, reaching up to 95%. The grain dimensions are from about 400 µm to 10 µm and below (Figure 3). A characteristic feature of all quartz grains is their irregular, angular shape. The quartz grains are accompanied by up to 5% grains of rock-forming minerals, mainly K-feldspars, Na-feldspars, apatite, zircon, rutile and amphibole, which have been variably subject to transformations resulting from firing at high temperatures (Figure 4 and Figures S3–S7). Most quartz grains reveal features typical of monocrystalline quartz (Figure 3). In plane-polarized light, most quartz grains are light-yellow or yellow-brown in colour. Only in the case of a few grains the intergrowths of two or more quartz grains were observed. There were also only a few grains composed of quartz intergrowths with other minerals, mostly with K-feldspars or Na-Ca-feldspars. In cross-polarized light, most grains were characterised by parallel extinction, only in a few grains the extinction is wave-type.

#### 3.2.2. Accessory Grains

The accessory grains are characterised by a much larger brightness in BSE images resulting from the larger average electron density. The grains were subject to analysis in order to determine: the chemical and mineralogical composition, the microstructure and the microtexture; as a result, 7 categories were distinguished (Figure 4 and Figures S3–S7 and Tables S1 and S2):

- category A—grains of accessory minerals with an unchanged texture (Figure 4). These are homogenous grains, with a structure and texture unchanged in course of firing at high temperatures. The only indication of these processes is the presence of a thin reaction zone at the boundary with grains having a different mineral composition (Figure 4c,f). The created zone plays the role of cement, a mass binding the mineral grains. The category includes grains of zircon, rutile, garnet, ilmenite, titanite, monazite, apatite, olivine, magnetite, and Na-, K- and Na-Ca-feldspars (Tables S1 and S2);
- category B—grains of rock-forming minerals, often with a partly changed texture (Figure S3). The category includes grains that contain even very small relicts of the primary mineral or those grains, in which its composition can be reconstructed based on the reaction products present. These are grains or rock-forming minerals with initial stages of melting or with large fragments of the mineral without a visible structural and textural reorganization. The transformation zone depends on the chemical composition of the mineral composing the grain and the surrounding grains/substances which have reacted with it during the firing process. The reaction area forms a coating, several to over 10 µm thick or extends within the grain along structural fractures but does not change its shape. This category includes minerals from the feldspar group with the composition of albite, sodium plagioclase, potassium feldspar, as well as biotite, epidote and amphibole (Tables S1 and S2);
- category C—grains of sulphides and oxides with an unchanged texture (Figure S4). The grains do not bear signs of structural-textural transformation on their surface or in their interior. Initial reactions with the surrounding grains (Figure S4f), colour change (changes of grey shade in BSE images), or melts on the mineral surface partly visible in the glass phase (Figure S4c) can be observed only in some grains. Some transformations are linked with the resistance of structural bonds of these minerals to high firing temperatures and the resulting high melting points. The category includes: ilmenite, cassiterite and sphalerite (Tables S1 and S2);
- category D—grains of sulphides with a partly changed texture (Figure S5). These are sulphide grains or aggregates of sulphide grains with a medium and strongly changed structure and texture, characterised by the presence of a reactive halo around the primary core of the mineral grain (Figure S5a). This zone extents for a few micrometres, which results from the impact of high firing temperatures on minerals with equally high melting points. Such textures appear around the grains of Co-Cu-Sn ore minerals (Tables S1 and S2);
- category E—grains of sulphides with a changed texture (Figure S5). These are single grains or their aggregates, which have been subject to almost complete structural and textural transformation. Oval metal oxide grains occur in the cover of a secondary silica melt (Figure S5b). The transformation includes partial or complete melting of sulphides and the appearance of drop-like accumulations of Cu, Ni and Co oxides (Tables S1 and S2);
- category F—grains of metal ores with a changed texture (Figure S6). These are ore grains with silicates or aluminosilicates, in which the primary structures and textures of mineral grains have not been preserved (Figure S6a,b) or are fragmentarily preserved (Figure S6c,d). Examples of such grains are the products of the reaction between galena and silicates and the resulting Pb silicates (Figure S6a,f) or secondary minerals belonging to Pb vanadates (Figure S6a,b, Tables S1 and S2);
- category G—lithoclast grains (Figure S7). The group includes large fragments of polymineral grains with dimensions up to 300 µm. These grains have well-preserved textures allowing for distinguishing the source rock lithology. Rock fragments with a characteristic interstitial texture are common (Figure S7a,c). Moreover, lithoclasts composed of sulphide minerals characterised by a variable grain size are quite common (Figure S7a,d). A continuous succession of sulphide grains from large lithoclasts with dimensions exceeding 300 µm to single sulphide grains with dimensions up to just over 10 µm can be observed in the analysed material. Sulphide lithoclasts are characterised by a strong degree of thermal transformations resulting from firing. An almost unchanged primary texture of the sulphide aggregates can be seen in only some larger grains (Figure S7d, Tables S1 and S2).

#### 3.2.3. Relationships of the Accessory Grains

Based on the analysis of about 390 gains, the group of common and accessory rock-forming minerals was identified: zircon, monazite, apatite, biotite, K, K-Na and Na-Ca-feldspars (plagioclases), epidote, amphibole, pyroxene, olivine, garnet, polymorphs of Al_2_SiO_5_, titanite, magnetite/hematite, ilmenite, TiO_2_ group minerals, Fe, Cu and Pb phosphates, Pb, Cu and As vanadates, barite and calcite (Table S4). The second group includes minerals representing sulphides, sulfosalts and arsenates, which were distinguished as ore minerals.

**Figure 5:**
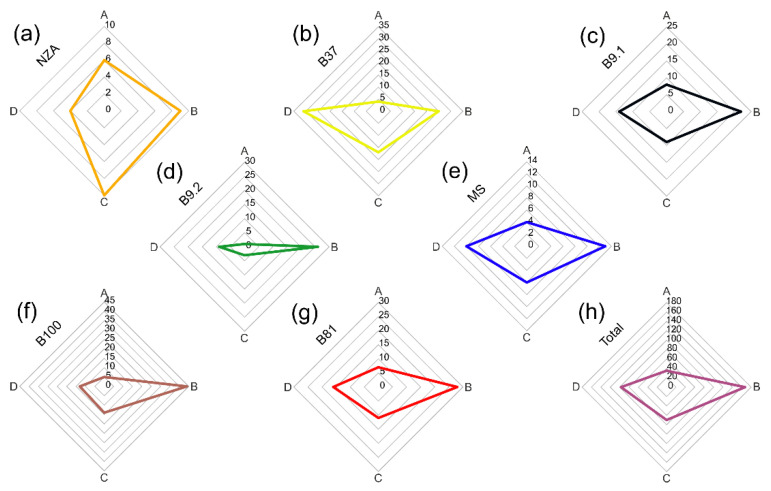
Diagrams showing the proportions of grains other than quartz determined in the faience body and glaze layer. Each axis shows grain number in a given group. (**a**–**g**) graphs with lab numbers and groups of minerals: A—accessory minerals (zircon, apatite, monazite), B—common rock-forming minerals, C—Fe-Ti oxides minerals (magnetite, hematite, ilmenite, TiO_2_ group minerals), D—ore minerals (sulphides, sulfosalts), (**h**) total graph—sum of all grains from graphs (**a**–**g**). See Table S2 for data from EDS analyses and grain mineralogy.

**Figure 6:**
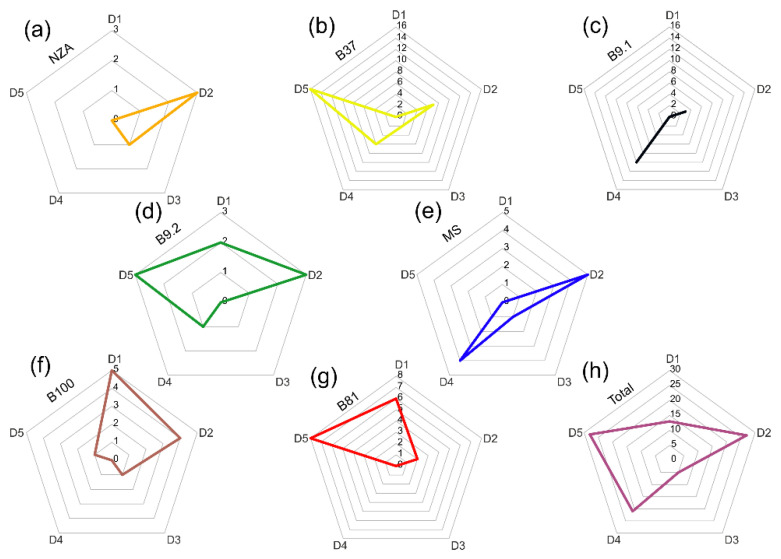
Diagrams showing the proportions of the distinguished subgroups from group D—associations of ore minerals in the form of ore grains from the faience body and glaze layer. Each axis shows grain number in a given subgroup. (**a**–**g**) graphs with lab numbers and subgroups: D1—cassiterite and polymetallic Sn-Co-Cu sulphides (Sn > 0.5 wt.%), D2—polymetallic Co-Cu-Ni sulphides (Sn < 0.5 wt.%), D3—polymetallic Cu dominated sulphides, traces of Bi, D4—polymetallic Pb-Sb-As-Cu sulphides and sulfosalts, D5—galena, sphalerite and polymetallic Zn-Pb dominant sulphides, (**h**) total graph—sum of all grains from graphs (**a**–**g**). See Table S2 for data from EDS analyses and grain mineralogy.

Minerals of accessory grains were subdivided into four main groups: A, B, C and D. Group D additionally comprises five subgroups: D1, D2, D3, D4 and D5. The contribution of grains from particular groups in the analysed accessory grains has been presented for each sample in the diagram (Figure 5 and Figure 6). The subdivision into the four main groups is as follows:

- group A—common accessory minerals represented by zircon, apatite and monazite;
- group B—common rock-forming minerals (amphibole, feldspars, epidote, titanite, garnet, olivine, biotite, pyroxene and Al_2_SiO_5_ polymorphs;
- group C—common Fe-Ti oxides: magnetite, hematite, ilmenite, and TiO_2_ group minerals;
- group D—sulphides representing ore minerals.

Diagram analysis points to the similarity of the mineral composition of accessory grains in all analysed samples (Figure 5). In each sample the contribution of the particular groups is similar, only sample NZA shows a larger contribution of minerals representing Fe-Ti oxides (group C). This opposite trend may be the result of a very small content of grains other than quartz present in this sample (only 29 grains were analysed).

All samples are characterised by the presence of sulphides, sulfosalts and arsenates representing ore minerals (group D), with large significance in the study of the quartz material provenance. Cassiterite, galena, sphalerite, chalcopyrite, pyrite and complex intergrowths of several minerals, strongly recrystallized under the influence of high firing temperatures were distinguished among the ore minerals. The largest number of grains from this group was noted in sample B37 (Figure 5b). The ore mineral group with a complex mineral composition was divided into 5 subgroups:

- subgroup D1—cassiterite intergrowths with ores and polymetallic Sn-Co-Cu sulphides, in which the Sn content in the products of sulphide reaction exceeds 0.5 wt.%;
- subgroup D2—polymetallic Co-Cu-Ni sulphides, in which the Sn content in the products of sulphide reaction does not exceed 0.5 wt.%;
- subgroup D3—polymetallic Cu-dominated sulphides, sometimes with a trace amount of Bi;
- subgroup D4—polymetallic Pb-Sb-As-Cu sulphides and sulfosalts;
- subgroup D5—galena, sphalerite and polymetallic Zn-Pb-dominant sulphides.

The number of grains representing ore minerals varies between the analysed samples (Figure 6). A total of 94 grains were analysed. The distribution of the number of grains composed of raw minerals is characterised by a larger variability in relation to the variability of the grains representing the rock-forming and accessory minerals (Figure 5). A group containing associations of minerals with Sn can be distinguished in samples B100, B9.2 and B81. The second, large group of grains significant for the interpretation of the origin of ore mineralization is represented by samples enriched in Co and Ni. The associations of ore minerals containing these elements in their chemical composition are present in all analysed samples, with their largest number documented in samples NZA and MS (Figure 6). The third group of grains of ore minerals contains Pb-Sb-S sulfosalts with a small admixture of minerals having As in their structure. This assemblage of ore grains was mainly observed in samples MS, B9.1 and B37. The last group, dominated by Zn and Pb sulphides prevails in sample B37, although a significant amount of sulphide grains belonging to this group was also observed in samples B9.2 and B81 (Figure 6). Cu and Fe sulphides occur in variable contents in most analysed samples.

#### 3.2.4. Interpretation

The analysed faience items are characterised by grain components dominated by coarse-crystalline quartz, a small number of minor and accessory mineral components and a small amount of the infilling mass (Figure 3 and Figure 4, Figures S3–S7). The main components of this mass are cementing substances formed during the firing processes, usually forming melts of metals and silicates as well as newly formed crystalline phases.

The shape of the quartz grains could not result from natural weathering and sedimentation processes of the siliciclastic material, and is rather the effect of crushing of larger blocks of monocrystalline quartz or large-sized quartz crystals (for details compare with Section 3.3.5). Wave type of extinction is a typical feature of quartz occurring in metamorphic rocks (gneisses, quartz-muscovite shales, metarenites). It is also commonly observed in plutonic rocks rich in quartz, e.g., granites or granodiorites. In turn the samples are dominated by quartz with parallel extinction (Figure 3), which points to its provenance from epigenetic veins largely composed of vein quartz and a small contribution of other mineral grains, mainly feldspars. These rocks often bear ore mineralization of variable intensity, from quantities of economic significance to accessory amounts. The quartz texture unequivocally indicates that it derives from veins containing small quantities of accessory minerals, including minerals representing Cu, Pb, Zn, Ni, Sn, Co, Te, As and Sb ores, which can be assigned to minerals with the highest potential to determine the provenance of the mineral raw material.

The grains representing common rock-forming and accessory minerals generally occur in various igneous and metamorphic rocks. Most analysed grains were subject to intense transformation under high temperatures during the firing process. The changes included structural-textural reorganization of the grains and change in the chemical composition caused by diffusion. Grains distinguished as category A have a relatively most unchanged chemical composition (Figure 4), being characterised by a chemical composition similar to the primary minerals occurring in the exploited rocks or applied tools. The second, extreme category are rather numerous grains of ore minerals, which during firing were subject to very intense modification of their structure and texture to the state when oxide melts rich in Fe, as well as Cu, Co, Ni, Sn, Zn, Pb, Bi, Sb and As were formed (Figure S5). The degree of thermal transformations does not allow for a precise identification of the mineral composition of the analysed grains. This is caused by the fact that during firing, numerous phases with a non-stoichiometric chemical composition were formed. However, it is possible to identify the primary mineral composition of the grains before the firing process. The identified mineral phases may be used in the provenance analysis of the studied raw material.

The group of ore minerals (Figure 6, subgroup D2) contains small fragments with dimensions from 60 to 30 µm, which are probably parts of larger clasts (Figure S5). Larger grains could form aggregates or intergrowths of several ore grains, which during firing entered into complex chemical reactions (Figure S6). Such small intergrowth forms of ore minerals are typical for assemblages of ore minerals occurring in various genetic types of mineralization [36,37]. Textures of these intergrowths are complex, with the presence of decay products of solid solutions and relicts of substitution textures even in very fine grains or ore aggregates.

Firing processes lead to significant changes of the chemical composition, being the effect of diffusion and oxidation, as well as reduction processes, which significantly changed the mineralogy of the primary grains. In some cases, the determination of the presence of primary mineral phases was possible based only on the analysis of the chemical composition and textures of the reaction products. In turn, the identification of mainly oxidation processes may point to the prevalent character of the atmosphere in the kiln, as already indicated by Zaremba et al., [18].

Most analysed grains are homogenous. They are fragments of larger mineral grains without inclusions. In the case of ore grains, a large part of the population was represented by intergrowths of several minerals or mineral grains with separation zones, which are often observed in assemblages of such minerals [36,37]. Complex grains, represented by aggregates of ore minerals were also observed in the analysed material (Figures S5 and S6), as well as lithoclasts with a texture typical for igneous rocks, e.g., clasts with an interstitial texture characteristic of dolerite rocks (Figure S7). The presence of a few fragments of lithoclasts in the analysed samples points to admixture of material derived from the rocks surrounding the exploited quartz veins. This fact explains the presence of other mineral grains, e.g., zircon, apatite, rutile, Ti-magnetite, amphibole and other grains, which are very rarely present in typical epigenetic vein quartz occurrences.

Some mineral grains have textural features allowing for them to be assigned to at least two distinguished textural categories of the grains, which may point to variable physical-chemical conditions of the firing process, as reflected in the thermal transformation of the grains. From among all the mentioned categories, the largest transformation with regard to texture and mineral composition have mineral grains belonging to sulphides and minerals containing large amounts of water in their structure, such as biotite or epidote. In the case of the latter, the firing processes could have caused partial melting of these minerals and induced the crystallization of new phases at temperature fall. Minerals stable in high temperatures, such as zircon, rutile, monazite, olivine, amphibole and pyroxene do not bear evident traces of transformation; only thin reaction zones with a changed and complex mineral composition are formed at the boundaries with grains characterised by a different composition.

Despite the lack of significant texture changes in grains of category A, the high temperature induced intense elemental diffusion, which influenced the chemical composition of the minerals. These processes intensified the development of reaction zones linking the neighbouring mineral grains. During temperature rise, a change in the Fe oxidation state could also take place. These changes are best seen on magnetite and ilmenite grains, in which oxidation of Fe leads to the formation of subtle zones visible in BSE images (Figure 4).

A characteristic feature of grains from category B is the presence of vast reaction zones caused by temperature increase, leading to the recrystallization or partial melting of large grain fragments, as well as development of reaction zones of different thickness around the grains.

Interstitial textures occurring in grains of category G are typical for dolerite rocks composed of plagioclase and clinopyroxene, as well as igneous rocks with diverse even-grained (Figure S7c) and porphyry-like (Figure S7b) textures. Grains with textures pointing to rapid crystallization from the silica solution represent another category of lithoclasts. Rapid crystallization confirms fast cooling of the kiln after reaching the maximal firing temperature, as indicated by Zaremba et al., [18]. These grains are built of amorphous glass, in which crystallize rock-forming minerals, including Fe oxides (Figure S7e). Such lithoclasts may represent fragments of earlier fired waste material after glass production or the glass frit. However, their relationship with volcanic rocks cannot be excluded. The last group assigned to the lithoclast category includes grains with complex textures typical for sedimentary rocks or with a structure typical for bone tissue (Figure S7f). Very often the samples that contained such grains have also a large number of products formed after the reaction of phosphorus with metals, mainly Pb. It should be emphasised that detritic apatite is usually only slightly changed and in most cases its development allows to assign it to category A, i.e., unchanged or slightly changed grains. In turn, the usage of bones as the source of P and Ca to the silica paste and glaze slurry was already mentioned by Zaremba et al., [18]. Lithoclast grains represent rock material from the surroundings of the exploited quartz veins. They may also derive from stone tools used for crushing and grinding. The intentional addition of such components to the silica paste seems highly improbable.

Temperatures exceeding 600 °C led to the formation of Pb silicates, thus the high content of Pb and Si in the EDS analyses may indirectly point to the occurrence of galena among the quartz grains. In turn, Fe-Ti oxides during firing underwent intense oxidation processes, which lead to the formation of hematite and anatase/brookite. The firing processes did not obliterate the primary textures of these oxides—titanomagnetites, as suggested by the homogenous ilmenite and inclusions of ilmenite lamellas in magnetite.

### 3.3. Morphometric and Geometric Analysis of the Quartz Grains

#### 3.3.1. Grain Size Composition

**Figure 7:**
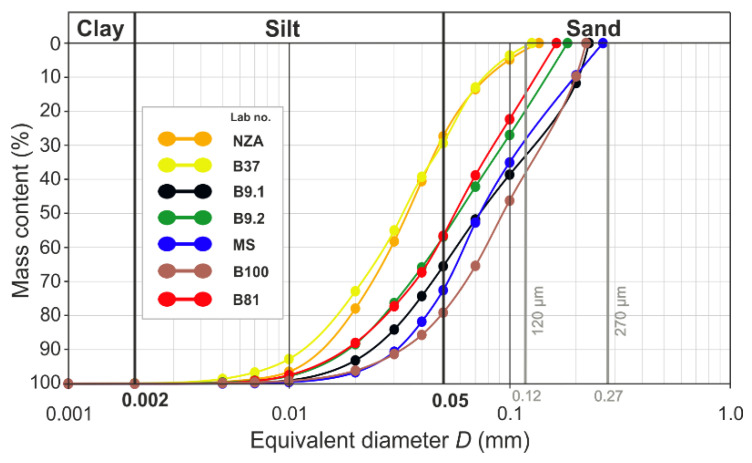
Quartz grain size distribution curves for the faience body layer. Grey lines (0.12 mm = 120 µm, 0.27 mm = 270 µm)—maxima of equivalent diameter (compare with Table S4).

**Figure 8:**
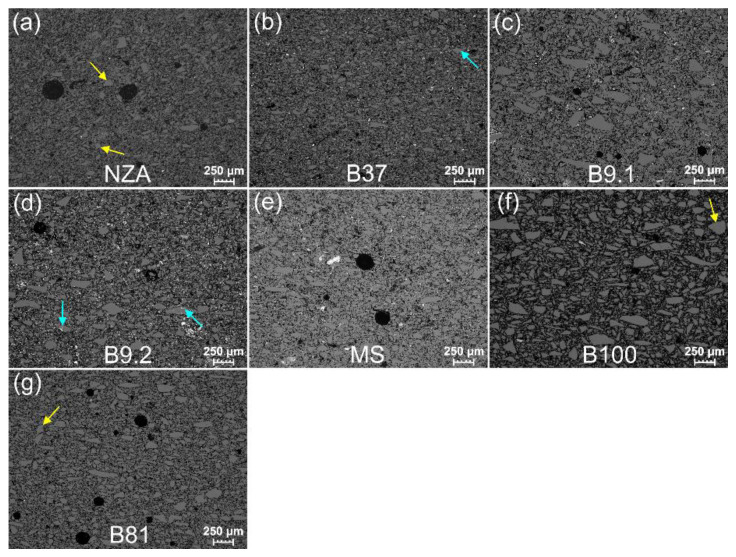
Microstructure of the faience body layer observed in a scanning electron microscope (SEM). (**a**–**g**) microphotographs with lab numbers viewed in the back-scattered electron mode (BSE), magnification ×100. Yellow arrows indicate isometric grains, blue arrows indicate grains with mineralised cracks.

**Figure 9:**
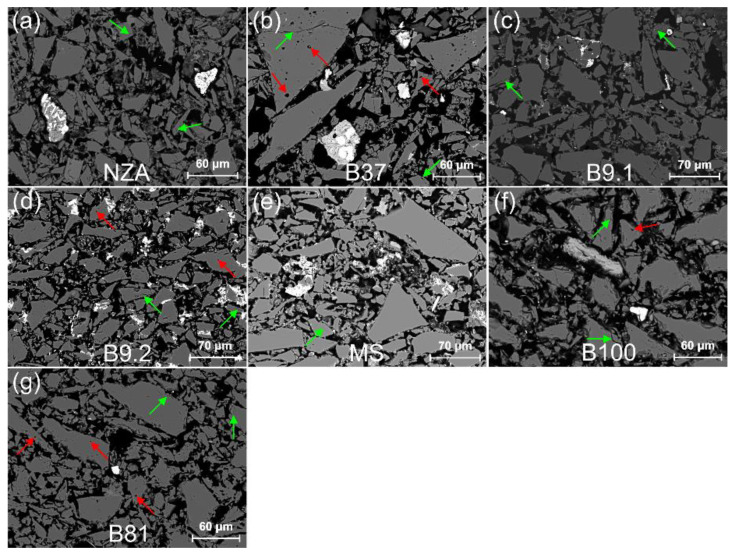
Microstructure of the faience body layer observed in a scanning electron microscope (SEM). (**a**–**g**) microphotographs with lab numbers viewed in the back-scattered electron mode (BSE), magnifications: (**c**–**e**) ×900, (**a**,**b**,**f**,**g**) ×1000. Green arrows indicate cracks in grains, red arrows indicate cavities.

The size of quartz grains in the core is variable (Figure S1 left photos, Figure 3, Figure 7, Figure 8 and Figure 9). The core of NZA and B37 is composed of grains below 50 μm (0.05 mm), whereas grains above 50 μm, maximally up to 200 μm (0.2 mm), are very rare (Table S3). In turn, B9.1 and MS contain more grains above 50 μm, and the content of grains above 100 μm (0.1 mm) is also larger. The core of B9.2 and B81 is characterised by a similar content of grains below 50 μm and grains in the range of 50–200 μm. The highest content of grains above 50 μm was noted in B100, particularly above 100 μm. Grains below 50 μm are very few in that sample. Detailed analysis of grain size confirms its variable composition (Figure 7 and Table S4). The core of NZA and B37 has over 70% of the silt fraction, whereas the sand fraction amounts to 30%. In B9.2 and B81 the content of the sand and silt fraction is similar and reaches 57% and 43%, respectively. In turn, B9.1, MS and B100 are dominated by the sand fraction (65.5–79%), whereas the silt fraction occurs at lowest levels (21–34.5%). A variable grain content in the particular size ranges has also been observed (Table S4). The core of B9.1, B9.2, MS, B100 and B81 is dominated by the sand fraction grains in the range of 0.07–0.2 mm (38–56%), whereas grains in the range of 0.05–0.02 mm prevail in the silt fraction (17–32%). Moreover, B9.1 and MS contain 10–12% grains in the range of 0.2–0.3 mm. In NZA and B37 the largest contributions have grains in the range of 0.07–0.01 mm (80–83%), which encompasses the lower range of the sand fraction and the upper range of the silt fraction.

The shape of the grain size curves is similar in the lower part and varies in the grain content (Figure 7). The upper part of the curves for NZA and B37 is bent, to gently reach the end of the curve, whereas the remaining curves are straight and reach the end at high angles. The maximal grain size varies from 0.12 mm (120 µm) for B37 to 0.27 mm (270 µm) for MS.

The variability of the size and content of grains in the core is documented by distribution histograms (Figure S8). The cores of NZA and B37, as well as B9.2 and B81 have the same grain size distribution to the range of 60–80 μm. The further course of the grain size distribution is monotonous with a higher contribution for B9.2 and B81. In turn B9.1, MS and B100 have a variable distribution. The most uneven distribution was noted in B100, which contains the largest number of grains above 90 μm, and in B9.1 the variability is lower above this size range. The smallest variability and lack of grains in the range of 180–260 μm was noted in the core of MS.

The morphometric parameters of the grains in the core change variably (Table 2). The range of changes in the relative area, total area and total perimeter is small, whereas the number of grains, average area, perimeter and diameter change in a wide range. At a low value of the number of grains and high average area (MS, B100, B9.1), the content of the sand fraction is twice higher than of the silt fraction. The increase in the number of grains and decrease in the average area (NZA and B37) is related with the twice smaller content of the sand fraction compared to the silt fraction.

#### 3.3.2. Grain Shape Composition

All samples show a similar composition with regard to grain shape (Figure S1 left photos, Figure 3, Figure 8 and Figure 9 and Table S3). Regardless of the grain size, anisometric and elongated shaped grains dominate. Grains with isometric shapes are few and are represented mostly by coarse grains (marked with yellow arrows in Figure 8a,f,g).

An analysis of the histograms of grain shape distribution shows the prevalence of anisometric grains (80.4–82.2%), with the smallest amount of elongated grains (0.7–2.1%), whereas isometric grains range between 16 and 19% (Figure S9 and Table 2). The anisometric shape range is dominated by grains with the average form index K_fav_ 0.35–0.55. The distribution is asymmetric with a maximum for K_fav_ = 0.40 and a rapid fall towards elongated grains. Towards the boundary line between the anisometric and isometric shape, the fall is gentle, and the continuation in the isometric grains is similar. The distribution for samples NZA and B37, as well as B9.2 and B81 is very similar and monotonous similarly as the grain size curves (compare Figure S9 and Figure 7). In turn, the distribution of the grain shape in samples B9.1 and B100 is non-uniform towards isometric grains, similarly as the grain size curves for coarse factions. The grain shape distribution in sample MS is different; compared to the irregular grain size curves it is monotonous.

The morphometric parameters of grain shape depending on their dimensions change variably (Table 2). For low values of N_s_ and P_t_ and high S_t_ (B9.1 and MS), the average form index K_fav_ is high, and the samples contain a low content of elongated and anisometric grains and a high content of isometric grains. Sample B100 displays a different dependency, as it has a low content of N_s_ and P_t_, and the S_t_ and average form index K_fav_ attain low values. This sample has a low content of grains with an isometric shape, and a high content of anisometric and elongated grains. In turn, sample B37 has high values of N_s_, P_t_, and S_t_, whereas the average form index K_fav_ attains the lowest value. This sample has the lowest content of isometric grains, and the highest—of anisometric and elongated grains. The remaining samples diverge from these relationships.

#### 3.3.3. Roundness and Sharpness

All samples have the largest content of very angular (VA) and angular (A) grains (Table S3 and Figure S1 left photos, Figure 3, Figure 8). Angular (A) grains prevail, whereas sub-angular (S-A) grains attain the lowest values. Only sample B37 contains VA and A grains, whereas sample MS has the largest content of S-A grains and the smallest content of VA grains. In samples B100 and NZA the grains usually have non-angular edges and corners, and jagged grains are the most common. In the remaining samples occur grains with sharp and rounded edges, as well as sharp and rounded corners, with no relationships between size and sharpness.

#### 3.3.4. Cracks and Cavities

The grains display numerous cracks which usually occur close to the edges and corners, and rarely pass across the entire grain (marked by green arrows in Figure 9). In samples NZA and B100, outline grain cracks are very numerous, whereas in sample MS they do not occur at all (Table S3 and Figure 8 and Figure 9). Some cracks across grain are filled with mineralization (marked with blue arrows in Figure 8b,d). Oval cavities with dimensions of maximally several micrometres (marked with red arrows in Figure 9b,d,g) occur in grains of all samples. The smallest number of oval cavities is visible in NZA and MS samples. Additionally, triangular cavities occur in samples B9.2, MS, B100 (marked with red arrow in Figure 9f) and B81 (Table S3).

#### 3.3.5. Microstructural and Microtextural Evidences of Quartz Grain Processing

- Grain size

A similar range of minimal and maximal grain sizes (from about 2 µm to nearly 300 µm) in all samples, as well as a similar distribution of their shapes results from the same crushing and grinding method (Table S4, Figure 7, Figure 9 and Figure S8). Approximate values of minimal dimensions may point to the technical possibilities of tools, which were used for quartz grinding. Rapid fall of the grain content below 10 μm may indicate the technical limits of the applied grinding tools (Figure 7). Below this value, very fine grains are the effect of thermal disintegration resulting from the high thermal expansion coefficient for quartz crystals [38]. The variable degree of grain disintegration resulting from the course of grain size curves is the result of exploitation of quartz veins with a variable character of mineralization (Figure 5, Figure 6 and Figure 7). The higher content of fine fractions in NZA and B37 results from a different type of vein mineralization, which were particularly rich in oxides, sulphides and sulfosalts (Figure 5). Such mineralization induced a higher degree of grinding, to separate very fine grains of metal ores from quartz. The contemporary specialists working on the quality of the exploited ore must have had knowledge on ore mineralization and processing. They must have also been able to macroscopically determine the degree of quartz grinding, which was indispensable for almost the entire retrieval of gold and other metals during washing. The lack of continuous grain sizes in the samples is caused by the duration and course of the grinding process and removal of some fractions during washing (Figure S8). After the source material was ground, quartz had much more grains with sizes larger than the ore minerals, because it usually has a much higher hardness. Therefore, it was more resistant to the friction forces during the grinding. In turn, the ore mineral grains had much smaller dimensions due to the lower hardness. Moreover, due to the much higher relative density, these minerals were more resistant to the forces of water flowing during the washing process and that is why they were subject to separation. Due to these processes, the fine fraction containing mainly the metal ore grains was diminished, as indicated by the grain size distribution for MS and B100 with a particularly high content of the sand fraction and a low content of the silt fraction (Table S4 and Figure 7).

The distinct upper limit of the grain dimensions from 0.12 to 0.27 mm (Figure 7) may point to a process of sifting coarser grains during grinding, for example by application of sieves, as suggested by Zaremba et al., [18] based on investigations of one sample. Sifting through very fine sieves is rather improbable due to the technical possibilities and the large scale of quartz material processing. The production of durable sieves allowing for the sifting of sharp and hard quartz grains was not possible in those times. In turn, mastering macroscopic analysis and organoleptic checking if the quartz material is sufficiently ground must have been commonly utilized by the ancient labourers and mine managers. The selection of coarser quartz grains which had not been ground could have taken place also during the washing process. The weight of such grains could withstand the velocity of the water stream resulting from the angle at which the water tables were installed. Such separated coarser material could be once more ground and washed.

After transportation from the mines in the Eastern Desert through the watercourse on the Nile and its supply to the Athribian workshops, the quartz material was subject to additional grinding depending on the needs. When the craftsmen required finer material, some parts of the source material were subject to further grinding. Different types of mortars and quern-stones have been found during the Tell Atrib excavations, and one of them, made of pink granite, with dimensions 21 × 26 × 7.5 cm, could be used to grind the quartz material. It is ellipsoidal in shape with an oval depression inside. The edges are rounded and the bottom side is smoothed with small cavity in the middle. Numerous grinding tools were also discovered, e.g., made from black stone with dimensions of about 6 × 6 × 4 cm (inventory numbers: TA/85/KT/96—mortar, TA/85/KT/135—grinding tool; [39]). The age of the surface layers, in which the tools have been found, was determined as probably Roman-Coptic, but the material was mixed [40]. Further grinding in these mortars did not affect the final grain shape distribution.

Analysis of the grain size in the core and the shape of faience vessels may indicate that source material with different grain size composition was used in the production depending on the type of vessel (compare different grain size composition between vessel NZA and other vessels, e.g., B100 in Table S4 and in Figure 8, compare fragment of vessel NZA with other vessel fragments in Figure 2 and shape reconstruction in [17]).

- Grain shape

The quartz grain shape being the result of crushing and grinding depends on the crystalline structure, which influences the physical and mechanical properties of the material. Quartz with a chemical formula SiO_2_ usually crystallizes in a hexagonal crystal system (high-temperature variety α) and a trigonal crystal system (low-temperature variety β). Crystals of this mineral usually have a columnar or acicular habit, rarely pseudo-regular. High hardness (7 in the Mohs scale) and lack of cleavage cause that quartz is a brittle mineral. The surface of crushed quartz attains a conchoidal fracture or is jagged, and the lustre is greasy. The perfect cleavage of some minerals induces crushing along even surfaces, and the resulting grains have regular shapes corresponding to the crystalline structure. In turn, quartz which only has a fracture breaks up along random and uneven surfaces. Therefore, crushed grains attain very irregular shapes. The second crucial factor which influences the grain shape is the grinding method and conditions, particularly the mechanisms of physical crushing and grinding, and the applied tools as well as the disintegration time. An additional factor influencing the grain shape is the input form of the source material used in the grinding and crushing, e.g., rounded grains of quartz sand. All these factors variably influence the final shape of the powdered quartz material.

Stone tools were used in the grinding process, which during the reciprocating action of the grinding stone on the dormant stone generated the mechanism of rolling, friction and shearing of grains between two surfaces. The occurrence of a variable shape of quartz grains, and their content and distribution (Figure 8 and Figure 9, Figure S9 and Table S3 and Table 2) confirm not only the influence of material properties of quartz on their shape, but also the method and conditions of crushing and grinding. Results indicate that the largest influence on the grain shapes had the grinding method. A large similarity in the distribution of grain shapes for all samples suggests the application of a similar grinding method and conditions. The prevalence of elongated and anisometric shapes with angular and jagged grains is caused by the action of friction and shear forces. The effect of rolling under load in the grinding process is visible to a lesser degree, as evidenced by a small content of isometric shapes and outline cracks (Table S3). Some cracks widened during the firing of faience items, when the crystals were subjected to thermal stress [18]; particularly the outline grain cracks (Table S3 and Figure 8 and Figure 9). An additional factor causing cracks to originate during crushing and grinding are numerous cavities in the grains, particularly triangular ones which are structural boundaries of the crystals (Table S3 and marked with a red arrow in Figure 9f). Surface roughness is also the result of rolling and occurs in the form of usually coarse, multifaceted grains with an isometric shape (arrowed in Figure 8a,f). Crushing and grinding caused by compression had the smallest contribution. Such mechanisms resulted in isometric grains and cracks across grains, which are relatively few (some are marked with green arrows in Figure 9). The duration of the grinding process did not significantly influence the grain shape, which is visible in the comparison between the variable size distribution and the lack of variability in shape distribution (compare Figure 7 and Figure S8 with Figure S9). The distinct microstructural and microtextural grain features clearly point to grinding of the quartz material in a dry mode without the addition of water, which is in line with the conclusions of Ulusoy et al., [41]. The presented material properties of quartz grains are observed in the same materials, which are subject to the same processes and with application of the same physical mechanisms, the process being facilitated with the application of modern tools [42,43].

The quartz grain shape influences the washing process of the ground material. Fine and medium-sized quartz grains of different shapes due to the low relative density of the mineral (2.65 Mg/m^3^) were washed down by the water stream along the oblique water table. In turn, the largest grains, often with elongated and anisometric shapes, were also washed down despite their large dimensions because when mounted transversely to the water current, they gave more resistance and had facilitated rolling.

- Relationships of morphometric parameters

Based on the matrix of the correlation coefficient, dependencies with high matching were determined (compare Table 2 with Figure S10). There is a very strong negative correlation between the number of grains and their average area and average diameter (Figure S10a,b). With the fall of S_av_ and D_av_ values, the Ns values rise in arithmetic progression. The course of the matching curves is almost parallel to the horizontal line of the chart attaining minimal values of the area (35 μm) and diameter (4.3 μm). The small number of large grains and the large number of small grains results from two factors. The first is the grinding process and its progress. Precise grinding of the material resulted in a larger number of smaller grains. The used tools allowed for very precise grinding of the source material allowing to reach the minimal value of about 10 μm, i.e., very fine silt (see D_av_ in Table 2). The second reason is linked with the recovery of metal ores during washing, which was described in detail for gold by Neesse [44]. The basic aim of excavating vein quartz and its crushing was obtaining the largest amount of metal ores, which induced the most precise grinding, followed by separating of such material. Washing caused separation of grains with a large weight and different sizes, mostly small ones. Almost pure quartz remained after this process, sometimes with a small content of metal ores concentrated on the grain surfaces, as observed in sample B9.2 (light fields in Figure 8d). The character and diversity of the quartz vein mineralization had a large influence on the final grinding of the raw material (Figure 8 and Figure 9). However, the distribution of the grain size in the ground material before washing is not known, whereas the variable distribution of size after washing confirms this influence.

There is a direct proportional relationship between the grain shape and size, which is represented by area and diameter (compare Table 2 with Figure S10c,d). K_fav_ increases with rising vales of S_av_ and D_av_. According to the correlation, smaller grains are more anisomeric and the larger ones are more isometric at increased grinding precision. However, the differences in the shape of smaller and larger grains are small, which points to the insignificant influence of the grinding precision on this parameter and the much larger influence of mechanisms and applied tools. Based on both correlations it can be concluded that the increased grinding progress results in the number of smaller grains with anisometric shapes. Similar results from studies made for the requirements of the mining industry were obtained by [41,42,43].

The distribution of grain shapes before washing was different, similarly to the distribution of their sizes, because the initial raw material was a mixture of metal ore grains and quartz. The ore grains which have material properties different than quartz, had a different shape distribution. In turn, the size distribution of ore grains and quartz was similar, with the prevalence of finer grains and with more isometric shapes for ore grains, as presented in some results for magnetite and quartz obtained by Dehghani et al., ([45], Figure 4, Figure 5 and Figure 6,8 and 9). Therefore, the grain size distribution for quartz did not change after washing, while the number of ore grains diminished, particularly with regard to small and medium grains, which were separated in the washing process (Figure 7). Due to the character of the quartz vein mineralization by different metals (Figure 5 and Figure 6), the reconstruction of the size and shape distribution of the raw material is difficult. The variable content of ore grains with different sizes and more isometric shapes caused a change in the course of grain size curves and distribution (Figure 7 and Figure S8) in its various parts, as well as a change in the grain shape distribution (Figure S9). In consequence, the position of points before washing in relation to that observed after washing would change on the correlation graphs (see example of reconstruction for one point in Figure S10c).

## 4. Discussion

### 4.1. Transformation of Minerals Resulting from the Firing Temperature

Based on XRD studies, α-cristobalite, a high-temperature variety of quartz, has been recognised in the faience body (Figure S2). In natural conditions this mineral occurs mostly in volcanic rocks, as well as in acid-sulphate-type hydrothermal alterations of volcanic rocks [46]. Cristobalite may also be formed in laboratory conditions, e.g., during the firing of a clay ceramic mass containing very fine quartz grains in temperatures exceeding 1100 °C [47]. The hydrothermal origin of the quartz veins used as the source material for the manufacturing of faience items, determined in course of our studies, excludes the natural origin of cristobalite in the analysed samples. In turn, the grain size composition of the body comprises very fine quartz grains even below 10 µm. The firing temperature of the analysed faience items has been determined as between 1050 and 1150 °C [18]. These facts evidence that cristobalite recovered in very small quantities is synthetic, being the result of the firing process. This has also been confirmed by the experimental studies of Kiefer and Allibert [48], who suggest that during refiring of ancient faience fragments, cristobalite appeared at 900 °C in the glaze, and at 1000 °C in the body of the vase fragment and bead. Further temperature rise resulted in increased contents of this mineral.

Various microscopic techniques are applied in archaeometric studies of ceramics with regard to the provenance of raw materials and the firing conditions, e.g., optical microscopy, scanning electron microscopy (SEM) coupled with chemical analysis at very small “spot” sizes (EDS, EPMA), and other supporting techniques, e.g., X-ray diffraction (XRD) or thermal analysis (TG/DTG-DTA). These techniques, after integration of analytical data, give appropriate final results. Gliozzo [49] and Zaremba et al., [18] emphasised the great importance of coupling image analysis with analysis of chemical or mineral composition, which are crucial but often overlooked. These authors point also to the need for combining several different methods in order to confirm results and obtain reliable outcomes. Detailed and multi proxy studies have also been commenced to the faience from ancient Egypt [18]. Due to the almost monomineral composition of the faience (over 95% of quartz in the body), many crystalline phases have not been identified in the XRD analyses, being modified during the firing process. The analysis has not supplied significant data for determining the provenance of the siliceous raw material. In turn, combining SEM image analysis with EDS chemical analysis at small volumes have allowed to identify numerous mineral phases in the body, i.e., accessory grains which have been subdivided into different categories (Table 3). This identification has provided critical information on the provenance of quartz. Some of the grains may be used as geothermometers, i.e., mineralogical thermometers, in future studies. After being heated to high temperatures, minerals are subject to many processes, such as melting or separation of solid solutions. Such phenomena supply data on the temperatures of the transformations, and in consequence may be used in the identification of the temperature at which the ceramics was fired. Detailed structural-textural analyses of variable transformed accessory grains supply precise data on the conditions at which faience objects were fired, the temperature and atmosphere. Among the observed processes that may be used for achieving these aims are: (1) separation of solid solutions, which may be observed e.g., in the case of Na and K feldspars, or sphalerite inclusions in chalcopyrite, (2) formation of silicate melts, and (3) formation of new phases (Table 3).

### 4.2. Origin of the Quartz

A series of accessory grains has been identified among the quartz grains in the faience material, which allowed for the characterization of the mineralization and determination of the provenance of the quartz material. The optical properties of quartz points to veins mineralized mainly with ore minerals. Minerals that serve as provenance proxies include sulphides and sulfosalts of Cu, Pb, Zn, Sn, Ni, Co, Te, As and Sb. The identified minerals include: arsenopyrite, chalcopyrite, stannite, cobaltite, nickeline, pyrrhotite, sphalerite, galena and cassiterite. Analysis of grains of the rock-forming minerals and lithoclasts indicates host rocks for the mineralized quartz veins. They include granitoids or altered granite rocks such as greisens. Part of the lithoclasts are fragments of dolerite rocks, which due to their mineralogical distinctness and high hardness represent material from the stone tools used for quartz disintegration.

Mineralization accompanying the quartz veins is comparable to the hydrothermal mineralization of the gold-bearing quartz veins on the Eastern Desert in Egypt [50]. The primary mineralization accompanying the gold-bearing quartz veins comprises mostly magnetite, ilmenite, pyrite, chalcopyrite, sphalerite and galena [51,52]. Moreover, cobaltine and tetrahedrite can also appear [53,54]. The lack of gold in the petrographic and mineralogical analyses in an optical microscope and chemical analysis in SEM–EDS was caused by fine grinding and removal of this metal from quartz, as well as the insufficient resolution of the analyses. A provenance from gold-bearing quartz veins, besides a hydrothermal origin and mineralization typical of such setting, is evidenced by the yellow-brown colour of quartz observed in optical microscopy, which is derived from metals, e.g., Au, dispersed in the quartz structure.

The epigenetic character of the quartz veins with regard to the surrounding rocks and the quartz-polymetallic sulphide type of mineralization evidences the provenance from two types of deposits, i.e., greisens and plutonic-hydrothermal ones. Quartz veins occur in both settings. Greisen deposits represent ores transitional to hydrothermal deposits and are related with ultra-acidic granites. Greisens developed in high temperatures of 500–600 °C in the topmost part of the granite massif. The mineralogical and chemical composition of the greisen ore deposits conforms with the composition of the accessory grains. Such origin is evidenced by EDS analyses, particularly the content of F, Cl, Cr and Mn, i.e., elements noted in all samples, as well as niobium and tantalum in samples NZA, B100, B81. Indicative of greisens are such elements as, e.g., wolfram in B100, molybdenum in NZA, B100 and B81, tin in all samples, and uranium in NZA and B100.

Plutonic-hydrothermal deposits are also linked with gold ores and polymetallic ores. The process of quartzitization in the veins took place at medium and low temperatures. The SiO_2_ content in such deposits reaches 90%. Chloritization took place together with postmagmatic alterations (tourmalinization), a process that was induced by the development of greisensation processes [55]. The chemical and mineral composition of these deposits has been confirmed in the composition of accessory grains, for instance, the high- and medium-temperature Pb-Sb-S mineralization which occurs in all samples, as well as such minerals as nickelin (NiAs), cobaltite (CoAsS), arsenopyrite (FeAsS), cassiterite (SnO_2_), stannite (Cu_2_FeSnS_4_), chalkopyrite (CuFeS_2_), sphalerite (ZnS), and galena (PbS). The presence of Ni-Co-Sn has been noted in all samples. Chlorites were not encountered in XRD analysis, as they must have been decomposed in temperatures close to 1000 °C during the firing process. However, their primary presence can be evidenced by elements such as Cr, Mn, Ni. The gold-bearing quartz veins are accompanied by pyrite (FeS_2_), sphalerite (ZnS), and galena (PbS), which have been identified in the accessory grains; Zn and Ag occur in all samples, while Pt was noted in NZA, B100 and B81.

The rich literature on the gold-bearing quartz veins from mines located in the Eastern Desert confirms the type of ore deposit and type of mineralization presented in the results [53,56,57].

### 4.3. Mining and Processing of Raw Material from Quartz Veins of the Eastern Desert

From the Early Dynastic Period in Egypt, the Eastern Desert was one of the most important exploitation areas of various mineral raw materials. The prospecting was focused on gold, silver, copper, lead and iron, which were obtained mainly from quartz veins in granitoid and granodiorite intrusive rocks [58,59]. In the case of gold, these ores are especially characterised by finely dispersed particles, measuring less than 10 µm, which are difficult to separate [44]. During the Old Kingdom and Middle Kingdom, vein quartz was ground by stone hammers. The rising number of mines during the New Kingdom combined with intense exploitation of newly discovered ore deposits induced technological and technical improvements and alterations. Grinding stones, for centuries used for grinding grains into flour, were applied for grinding of the raw material [58,60]. Thus, the quartz material was ground into fine powder, and then washed to separate the metal ore particles. In the Ptolemaic Period, subsequent technological modifications were linked with adaptation of new types of mills and grinding stones. New techniques imported from Greece to Egypt using perfectly circular large mills and concave shaped mill stones allowed for much more effective crushing and grinding of the quartz material [58,61]. Mills and stone mortars discovered in archaeological sites around the mines are made from local hard rocks. As mentioned by Diodorus, according to Agatharchides of Cnidus (mid-2nd century BC), recovered lumps of gold-bearing quartz were crushed into smaller fragments until the grains reached the size of vetch seeds, i.e., about 5 mm. Such ground material was ground in mills or mortars into fine powder, which was then washed with water on inclined tables in order to separate gold from quartz [62]. The washing tables were also made of local material, usually from slate rocks forming flat stones. The base of the rectangular tables was 2.2–4 m in length and 40–60 cm in width, while the inclination was at 15–20° and had a maximal height of 80–100 cm. Such ramp made of loose material was laid with flat stones, and the construction was additionally sealed with mortar. At the base of the ramp was installed a collecting basin, 60–80 cm in size, also sealed with flat stones and mortar, and with a back flow channel, which allowed to retrieve water during the washing process [58]. The remains of this process are numerous tailing heaps of fine-grained quartz, which have not been precisely analysed yet. Brief remarks on the material from the heaps have been published for eight mines exploiting in different historical periods. The tailing heaps are mostly pink to reddish in colour and are composed of angular quartz grains. Additionally, the heap from the Umm Garaiyat mine contains only milky and translucent quartz grains [58,63]. In turn, in the material from tailing heaps of the Samut North mine dated at the Ptolemaic Period, the quartz grain sizes are from 0.05 to 0.1 mm (50–100 µm) [64]. Archaeological discoveries in the gold mines confirms Agatharchides’ chain of operation that led from ore to gold particles.

**Figure 10:**
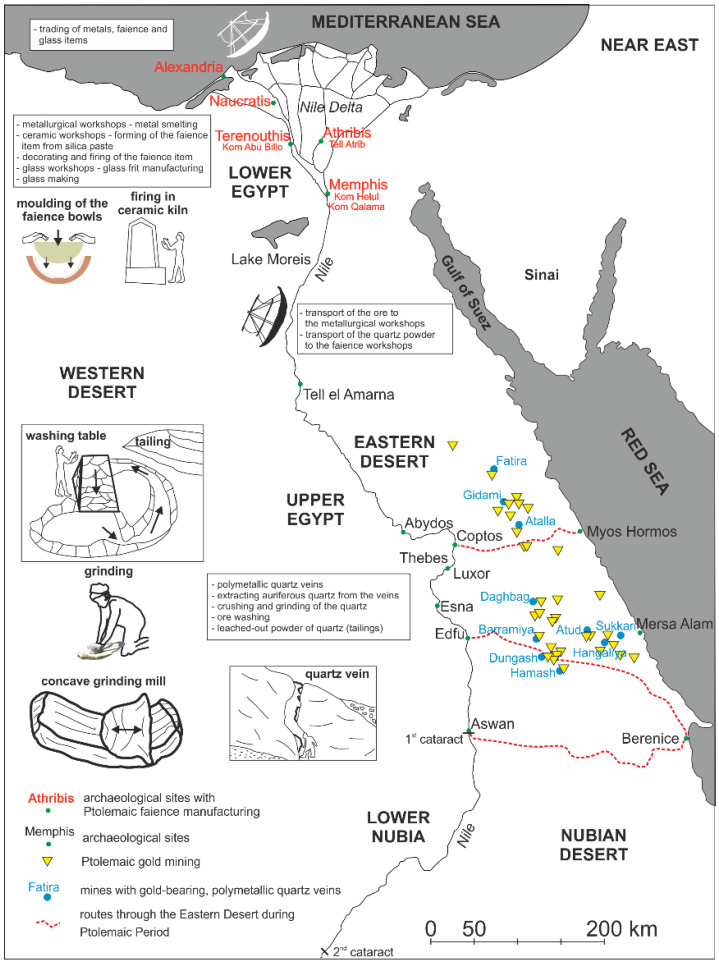
Provenance of raw materials and their processing for the manufacturing of Ptolemaic faience items. Gold mining in the Eastern Desert and Ptolemaic faience workshops in the Nile delta: location of archaeological sites with Ptolemaic faience manufacturing, Ptolemaic gold mines with polymetallic mineralization and possible mining sites of the raw materials used in Athribian workshops. Ptolemaic gold mining according to Klemm et al., [58] and routes through the Eastern Desert during the Ptolemaic Period according to Sidebotham [65].

The obtained metal ore concentrates were transported to metallurgical workshops, where they were smelted to “pure” metal. Many of these workshops were located in the Nile Delta, where there was plenty of fuel for the furnaces. In turn, quartz after washing remained on the heaps close to the washing tables. Tailing heaps of different sizes were observed, whereas in some sites there are no tailing heap fragments or even their traces. According to Klemm and Klemm [63], the cause of these lacks is the activity of erosional processes. In the Eastern Desert, erosional processes are very slow in geological time and they could not have removed the material from the tailing heaps completely or partly without leaving any traces. Additionally, the most frequently observed pink to reddish colour of the tailing heaps is different from the milky-white and blue-grey colour of the vein quartz. This fact may point to the lack of tailing heaps with quartz of a neutral colour. In turn, weathering processes are much faster in this area and could have changed the colour to pink and red in those parts of the tailing heaps where the quartz had lower quality, and was polluted with metal compounds and other minerals, e.g., from the vein borders where it contacted with the surrounding rocks. The lack of heaps with quartz from the pure parts of the veins suggest removal of the material from the mines and its usage for other purposes (Figure 10).

Quartz heaps in mines represent waste material with regard to the exploited metals, but with features of raw material that enabled its usage in the production of faience and glass items. The raw material was homogenous, purified, with an almost neutral colour, well ground (to fine powder) and could contain only a few remains of various metals and other minerals (observed in microscale in Figure 5 and Figure 6). These additives could have a favourable influence on the manufacturing process by playing the role of flux during firing or melting. The usage of quartz raw material with good technical parameters in workshops dealing with faience or glass production gave also new possibilities of manufacture and could induce technological changes and development of these branches of craftsmanship.

### 4.4. From Gold Mining in the Eastern Desert to Faience Manufacturing in Tell Atrib

Acquiring large amounts of gold and other metals from the mines of the Ptolemaic Period on the Eastern Desert was linked with intense mining and processing of quartz veins. Quartz grinding was accomplished mainly by concave mills; in this case the quartz grains usually had anisometric and elongated shapes. In turn, in the Roman and Arabic periods, grinding was accomplished by rotary querns (rotary or round mills). The application of such techniques results in the prevalence of isometric shapes among the quartz grains ([41] and interpretation in Section 3.3.5). However, there is a lack of detailed data from mine tailings and from investigations performed on faience objects (e.g., [7,10,12,13,63]).

Rotary mills used in gold mines were more effective and gave more fine-grained quartz than concave mills [41], reducing the gold extraction process by 1/3 with regard to the earlier used technology [58]. Macroscopic analysis of the core of faience items from the Roman Period shows a coarse-grained composition with single sand grains [10]. SEM-EDS analysis of the grains of a faience body from Memphis also suggest the application of quartz sand as the raw material [15]. This may indicate the usage of a different raw material in the workshops compared to the finely ground quartz from the gold mines of the Eastern Desert. Such local raw material could be sediments of the Nile delta, sands or washed alluvial muds, or aeolian sands from the Western Desert. In turn, the studies of Welc [17] suggest that the faience items from Tell Atrib were produced from material acquired from quartz veins between the mid-3rd century BC and the 1st century AD, with a declining quality of faience objects at that time. This must have resulted from the smaller requirements of the purchasers who searched for cheaper products. Such a situation could have been influenced by the changing political-economic conditions in Egypt. Quartz from the mines in the Eastern Desert was a very good raw material for faience manufacturing but it must have been also more expensive compared to the local source, which was almost free of charge. In turn, vein quartz gave the possibility of manufacturing high-class relief decorated vessels for demanding and rich customers. Due to the application of cheaper raw material and technologies, the Memphis workshops were capable of producing faience objects on a larger scale and for less demanding consumers.

Mines from earlier periods, particularly from the Ptolemaic Period and New Kingdom, were often used in the Roman-Byzantine times [63]. The common usage of rotary mills in this period was linked not only with their higher effectiveness, but also with the utilization of partly exploited deposits that had a lower content of Au and other metals. This could have imposed very fine grinding in order to separate the metal ore grains during the washing process.

The manufacturing of faience objects in the Roman Period that had a different quality from those from the Ptolemaic Period might have been caused by other reasons. The increase in production and quality of glass objects [66] must have resulted in their rising role in every-day life and caused higher competition with regard to faience items. Glass products were also of better material quality compared to faience items, and the technology gave better possibilities of obtaining more practical and exclusive products. The glass objects could have been manufactured with application of quartz from the mines in the Eastern Desert as a source fulfilling high pureness standards. The high price of this raw material could have been compensated by a good price of the glass items on the market. In the Roman Period, pure quartz sand recovered from the Nile delta was used for glass manufacturing, which allowed for the production of high-quality items. Such objects made of antimony glass known as Alexandrian glass have been discovered in the Jerash site in Jordan, and the provenance of the raw material from the Nile delta was confirmed by Barfod et al., [67]. These investigations evidence that determining the provenance of raw material is possible also in cases of their complete transformation.

In the Roman Period, gold mining in Egypt started to play a less significant role due to its discovery in other regions in the Roman Empire, mainly in the British Isles and Iberian Peninsula, where the gold deposits were more accessible and richer [68,69].

The lack of gold particles in the analysed samples of the faience products results from a number of causes. One of them is the precise grinding of the recovered quartz into fine powder, followed by washing. Throughout the course of these processes, the metal ore grains, particularly gold, were separated from quartz in water flowing down the washing table. For example, Au particles with dimensions of about 10 µm had the same weight as the quartz grains with dimensions of about 100 µm, which is a 10-wise mass difference. These physical relationships determine the grain-size distribution and their maximal sizes (Figure 7 and Figure S8). Therefore, the entire amount of gold was very precisely separated from the remaining grains. Trace quantities of Au could occur as very fine inclusions in the quartz grains, but confirming this assumption requires detailed investigations and chemical analysis at high resolution levels, whereas EDS analyses do not assure such precision. Another reason may be the character of Au occurrence in the vein quartz deposits. Gold is not evenly distributed in the rock but occurs in larger but patchy concentrations [70]. Therefore, the recovery of quartz from a tailing containing more gold is statistically highly improbable in a thin section. The ICP-OES analysis performed on the MS sample has confirmed the content of niobium at 27 μg/L, lithium at 1.3 μg/L and gold at 23 ppb, which was on detection limit. Such Au content in quartz points to its gold-bearing character. Similar low content of gold were recorded from the Atalla’s quartz veins [71]. The presence of lithium indicates for greisen-type tourmaline-bearing quartz veins with gold and copper. An additional proof of such quartz origin is its brown-yellow colour observed in transmitted and reflected light microscopy, which results from the subnanometric accumulations of various metals, including Au, dispersed in the crystals. The Ag and Cu content in the accessory grains of almost all analysed samples points to the usage of gold-bearing quartz veins for the manufacturing of faience (Tables S3 and S4). The mineralisation of such veins contains 5–10% Ag and the same amount of Cu in relation to the Au content [72]. A similar proxy indicating the usage of quartz from gold-bearing veins of the Eastern Desert is the Pt content [73], identified in the grains of some samples. EDS analyses of the chemical composition of gold used for decorating stuccos from the Ptolemaic Period in Tell Atrib have revealed the presence of Au and Cu, as well as minor quantities of Fe. This may suggest the contact of the craftsmen from Tell Atrib with suppliers of Au ore from the mines in the Eastern Desert. Such contacts could have been used also for the supply of quartz material to the same workshops that produced faience objects in Tell Atrib.

Out of over 30 mining regions from the Ptolemaic Period, based on the analysis of the geological structure, i.e., polymetallic quartz veins of plutonic-hydrothermal and greisenisation origin, 10 sites from which the quartz used for the manufacturing of faience objects in Athribian workshops have been selected. Geological and petrographic-mineralogical data from the mines were compared with the chemical and mineral composition of the accessory grains identified in the faience objects. The selected mines include (see Figure 10, no. of gold mine according to Klemm and Klemm [63]): Fatira (Abu Zawal) (5.2.2), Gidami (5.2.13), Atalla (5.2.24), Daghbag I–IV (5.3.12.1–5.3.12.3), Barramiya (5.4.5), Atud (5.4.6), Sukkari (5.4.12), Dungash (5.4.22), Hangaliya (5.4.28), and Hamash (5.4.34). The most important minerals, considered as proxies, include tetrahedrite belonging to Cu sulfosalts and cobaltite belonging to As sulfosalts [53,56,57,63]. Ni and Co played a crucial role in the selection as admixtures, as well as sphalerite, pyrite and arsenopyrite [51,52,63,72,74].

## Figures and Tables

**Table 1:** Examined types of faience bowls from Tell Atrib. Order of samples (lab no.) applied in other figures and tables.

Cat. no. ^a^	Bowl Type		Dating ^a^	Lab no. ^d^	Characteristics of the Faience Fragment (Compare Figure 2 and Figure S1)	
	Cat. ^a^	Others			Fragment	Material
195	A-IV	T5.2 ^b^	mid-3rd century BC	**NZA**	rim and upper body	Body: 3–6 mm thick, yellow, 2.5 Y 9/2 ^f^ Glaze: ~0.3 mm thick, smooth, blue-green, 2.5 BG 8/2
37	B.1	T1 ^c^	2nd half of 3rd century BC	**B37**	rim and upper body	Body: 5–6 mm thick, yellow, 2.5 Y 9/3 Glaze: ~0.1 mm thick, worn, blue-green, 10 BG 9/2
9	B.1	T1 ^c^	end (?) of 3rd century BC	**B9.1 ^e^**	lower body	Body: 3–4 mm thick, yellow-red, 10 Y 9/1 Glaze: ~0.15 mm thick, glossy, blue, 2.5 B 8/2
9	B.1	T1 ^c^	end (?) of 3rd century BC	**B9.2 ^e^**	lower body	Body: 3–6 mm thick, yellow, 5 Y 9/1 Glaze: 0.2–0.4 mm thick, glossy, blue-green, 5 BG 9/2
4	B.1	T1 ^c^	end (?) of 3rd century BC	**MS**	base and lower body	Body: 5–6 mm thick, yellow, 2.5 Y 9/2 Glaze: ~0.3 mm thick, glossy, smooth, blue-green, 2.5 BG 9/2
100	B.5	T2.1^c^	end of 3rd century BC or 1st half of 2nd century BC	**B100**	rim and upper body	Body: 5–7 mm thick, yellow-red, 10 YR 9/3 Glaze: 0.15–0.2 mm thick, matt, blue-green, 7.5 BG 9/2
81	B.4.1	T2.3d ^c^	1st half of 2nd century BC	**B81**	rim and upper body	Body: 3–5 mm of thick, yellow-red, 10 YR 9/1 Glaze: ~0.1 mm thick, worn, blue-green, 10 BG 9/2

^a^ catalogue number, type and dating according to Welc [17]: A-IV—Achaemenid-Influence Vessel, B.1—Relief-decorated hemispherical bowls, B.4.1—Relief-decorated bowls, B.5—Relief-decorated deep bowls; ^b^ type according to Nenna [15]: T5.2 Achaemenid inspired shapes: Spherical bowl with cylindrical neck; ^c^ types according to Nenna and Seif el-Din [10]: T1—Shallow hemispherical bowls, T2.1—Deep bowl with flared lip, T2.3d—Deep bowl with tapering walls and geometric decorations covering the entire body (exterior); ^d^ laboratory number used in the text and graphics of the paper; ^e^ catalogue number the same (9) but laboratory number is different; two fragments come from different faience objects—compare Welc ([16], p. 264) and Figure 2c,d and Figure S1e–h; ^f^ symbols of colour according to the Munsell Colour Chart.

**Table 2:** Morphometric parameters of the quartz grains from the faience body layer. Note values of parameters and compare with the same parameters in Table S4 and Figure 8, Figures S8 and S9.

Grains Parameters	Lab No.						
	NZA	B37	B9.1	B9.2	MS	B100	B81
Relative area *s* (%)	55.0	60.9	62.8	64.0	63.2	55.7	66.2
Number *N_s_* × 10^3^ (−)	51	111	42	65	18	37	42
Total area *S_t_* × 10^3^ (µm^2^)	3638	3937	4141	4199	4233	3674	4418
Average area *S_av_* (µm^2^)	71	35	99	65	237	101	106
Total perimeter *P_t_* × 10^3^ (µm)	1889	2608	1700	2020	1332	1341	1863
Average perimeter *P_av_* (µm)	37	24	41	31	75	37	45
Average diameter *D_av_* (µm)	6.1	4.3	6.0	5.2	10.0	5.6	7.5
Sand Ø > 50 µm (%)	27.4	29.4	65.5	56.9	72.6	79.1	56.6
Silt 2 < Ø < 50 µm (%)	72.6	70.5	34.5	43.1	27.4	20.9	43.4
Clay Ø < 2 µm (%)	0	0.1	0	0	0	0	0
Average form index *K_fav_* (−)	0.477	0.456	0.481	0.475	0.488	0.468	0.477
Elongated grains (%)	1.0	2.1	1.0	1.5	0.7	1.4	1.4
Anisometric grains (%)	81.6	82.2	80.8	80.7	80.7	82.0	80.4
Isometric grains (%)	17.4	15.7	18.2	17.8	18.6	16.6	18.2

**Table 3:** Provenance of the accessory grains from the faience body and glaze layer.

Accessory Grains		Primary Mineral, Rock	Degree, Character of Transformation	Secondary Mineral, Alloy	Indicator of Raw Materials Provenance	Firing Temperature Indicators [°C]
Category	Change in Texture					
A accessory minerals	unchanged	zircon, rutile, garnet, ilmenite, titanite, monazite, apatite, olivine, magnetite, Na-, K- and Na-Ca feldspars	very low, thick layer of reaction	-	Host rocks: granodiorite, monzogranite, porphyry	-
B rock-forming minerals	partially changed	albite, Na plagioclase, K feldspar, biotite, epidote, amphibole	low, initial melted	-	Host rocks, contact zone of hydrothermal vein: granodiorite, monzogranite,	Formation of perthites
C sulphurs and oxides	unchanged	ilmenite, cassiterite, sphalerite	very low, without change	-	High and mid-temperature hydrothermal veins	-
D sulphurs	partially changed	Co-Cu-Sn ores	low, thick layer of reaction (halo)	-	High and mid-temperature hydrothermal veins	?
E sulphurs	changed	sulphurs	medium and high,partially or completely melted	drops of Cu, Ni, Co oxides in silica alloy	High and mid-temperature hydrothermal vein	Exsolution textures: chalcopyrite in sphalerite ca. 550 °C
F ores and sulfosalts	changed	Pb ores (galena), sulfosalts Pb-Sb-S with silicates or aluminosilicates	High, completely melted	Pb silicates	Low-temperature hydrothermal vein	PbS-SiO_2_ alloy forming at 775 °C
G lithoclasts (lithic fragments)	unchanged, change, intersertal, porphyry	igneous and dolerite rocks, volcanic effusive rocks, sulphurs	medium and high,partially or completely melted	polymineral sulphurs alloy, Pb-P alloy Fe oxides	Surrounding rocks—source for stone tools	Dolerite rock melting starts at ca. 800 °C

## Supplementary Materials

The following supporting information can be downloaded at: https://www.mdpi.com/article/10.3390/ma15186251/s1, Figure S1: Microstructure of the body and glaze layer observed under a light microscope. (a–n) pairs of microphotographs with lab numbers: left side—fresh fracture, right side—glaze surface. Symbol of colour according to the Munsell Colour Chart (compare Table 1 and Figure 2); Figure S2: X-ray Powder Diffraction Analysis (XRD) of the faience body. (a) summary of the diffractograms with lab numbers, (b) magnified part of the diffractogram B100. Reflections: Q—quartz, C—calcite, Cr—cristobalite, !—“apparent” reflection linked to Kβ, X-Ray component (originating from [011] plane of quartz), ?—reflections which could not be reliably identified. For better clarity of the figure, reflections Q and ! were not marked above each diffractogram. The annotations have been made only above the diffractogram of sample NZA; Figure S3: Accessory grains of the faience body and glaze layer observed in a scanning electron microscope (SEM). Category B—rock-forming minerals, often with a partly changed texture. (a–f) microphotographs with lab numbers viewed in the back-scattered electron mode (BSE). Magnifications: (a) ×3000, (b) ×2000, (c) ×1000, (d) ×2500, (e) ×4000, (f) ×2000. Location of EDS analyses marked with blue numbers and dots (see Tables S1 and S2 for elemental and mineral composition); Figure S4: Accessory grains of the faience body and glaze layer observed in a scanning electron microscope (SEM). Category C—sulphides and oxides with an unchanged texture. (a–f) microphotographs with lab numbers viewed in the back-scattered electron mode (BSE). Magnifications: (a) ×3000, (b) ×2500, (c) ×2000, (d) ×1500, (e) ×2000, (f) ×3000. Location of EDS analyses marked with blue numbers and dots (see Tables S1 and S2 for elemental and mineral composition); Figure S5: Accessory grains of the faience body and glaze layer observed in a scanning electron microscope (SEM). Categories D and E—sulphides with a partly changed and changed texture. (a–f) microphotographs with lab numbers viewed in the back-scattered electron mode (BSE). Magnifications: (a) ×2000, (b) ×3500, (c) ×1700, (d) ×3000, (e) ×2000, (f) ×2000. Location of EDS analyses marked with blue numbers and dots (see Tables S1 and S2 for elemental and mineral composition); Figure S6: Accessory grains of the faience body and glaze layer observed in a scanning electron microscope (SEM). Category F—ores with a changed texture. (a–f) microphotographs with lab numbers viewed in the back-scattered electron mode (BSE). Magnifications: (a) ×1500, (b) ×2000, (c) ×1500, (d) ×2500, (e) ×5000, (f) ×2000. Location of EDS analyses marked with blue numbers and dots (see Tables S1 and S2 for elemental and mineral composition); Figure S7: Accessory grains of the faience body and glaze layer observed in a scanning electron microscope (SEM). Category G—lithoclasts (lithic fragments). (a–f) microphotographs with lab numbers viewed in the back-scattered electron mode (BSE). Magnifications: (a) ×2500, (b) ×1500, (c) ×1600, (d) ×3000, (e) ×2500, (f) ×1500. Location of EDS analyses marked with blue numbers and dots (see Tables S1 and S2 for elemental and mineral composition); Figure S8: Quartz grain size distribution of the faience body layer. (a–g) histograms of grain size distribution with lab numbers. Mass content = *M_i_/M*, where *M_i_*—mass of grains within the “*i*” interval, M—total mass of all grains. Compare with Figure 7 and Table S4; Figure S9: Quartz grain shape distribution of the faience body layer. (a–g) histograms of the grain shape distribution with lab numbers. Grains with different shapes were distinguished based on the distribution of the form index *K_f_*: isometric a/b < 1.5, anisometric 1.5 < a/b < 10 and elongated a/b > 10 (a, b—extreme grain dimensions). Probability density of grains *Ph_i_ = N_i_/Nxl*, where *Ni*—number of grains within the “*i*” interval, *N*—total number of grains, *l*—length of the interval; Figure S10: Relationships between morphometric parameters of the quartz grains from the faience body layer. (a–d) correlations between selected parameters from Table 2. Dots marked with the same colour as the lines and lab numbers in Figure 7 and Table 2 and Table S4. Table S1: Elemental composition of the accessory grains from the faience body and glaze layer. See Figure 4, Figures S3–S7 for location of EDS analyses; Table S2: Data base of SEM-EDS analyses of the accessory grains from the faience body and glaze layer; Table S3: Microstructural and microtextural data of the quartz grains from the faience body layer (compare with images in Figure S1, Figure 8 and Figure 9); Table S4: Quartz grain size composition of the faience body layer. Lab numbers marked with the same colour as the lines in Figure 7 (note value of mass content for different size ranges and compare with the same data).
