# Supplementary material for: Source of Raw Materials and Its Processing for the Manufacturing of Ptolemaic Faience Bowls from Tell Atrib (Nile Delta, Egypt)
Source: Materials (Basel). 2022 Sep 8;15(18):6251. doi: 10.3390/ma15186251 (PMC9501641; doi:10.3390/ma15186251)
Supplement: Supplementary file 1 [file materials-15-06251-s001.zip › Supplementary materials_revised/Supplementary materials_captions_revised.pdf]

Figure S1. Microstructure of the body and glaze layer observed under a light microscope. (a–n) pairs of microphotographs with lab numbers: left side – fresh fracture, right side – glaze surface. Symbol of colour according to the Munsell Colour Chart (compare Table 1 and Figure 2);

Figure S2. X-ray Powder Diffraction Analysis (XRD) of the faience body. (a) summary of the diffractograms with lab numbers, (b) magnified part of the diffractogram B100. Reflections: Q – quartz, C – calcite, Cr – cristobalite, ! – "apparent" reflection linked to Kβ, X-Ray component (originating from [011] plane of quartz), ? – reflections which could not be reliably identified. For better clarity of the figure, reflections Q and ! were not marked above each diffractogram. The annotations have been made only above the diffractogram of sample NZA;

Figure S3. Accessory grains of the faience body and glaze layer observed in a scanning electron microscope (SEM). Category B - rock-forming minerals, often with a partly changed texture. (a–f) microphotographs with lab numbers viewed in the back-scattered electron mode (BSE). Magnifications: (a) x3000, (b) x2000, (c) x1000, (d) x2500, (e) x4000, (f) x2000. Location of EDS analyses marked with blue numbers and dots (see Tables S1, S2 for elemental and mineral composition);

Figure S4. Accessory grains of the faience body and glaze layer observed in a scanning electron microscope (SEM). Category C - sulphides and oxides with an unchanged texture. (a–f) microphotographs with lab numbers viewed in the back-scattered electron mode (BSE). Magnifications: (a) x3000, (b) x2500, (c) x2000, (d) x1500, (e) x2000, (f) x3000. Location of EDS analyses marked with blue numbers and dots (see Tables S1, S2 for elemental and mineral composition);

Figure S5. Accessory grains of the faience body and glaze layer observed in a scanning electron microscope (SEM). Categories D and E – sulphides with a partly changed and changed texture. (a–f) microphotographs with lab numbers viewed in the back-scattered electron mode (BSE). Magnifications: (a) x2000, (b) x3500, (c) x1700, (d) x3000, (e) x2000, (f) x2000. Location of EDS analyses marked with blue numbers and dots (see Tables S1, S2 for elemental and mineral composition);

Figure S6. Accessory grains of the faience body and glaze layer observed in a scanning electron microscope (SEM). Category F - ores with a changed texture. (a–f) microphotographs with lab numbers viewed in the back-scattered electron mode (BSE). Magnifications: (a) x1500, (b) x2000, (c) x1500, (d) x2500, (e) x5000, (f) x2000. Location of EDS analyses marked with blue numbers and dots (see Tables S1, S2 for elemental and mineral composition);

Figure S7. Accessory grains of the faience body and glaze layer observed in a scanning electron microscope (SEM). Category G - lithoclasts (lithic fragments). (a–f) microphotographs with lab numbers viewed in the back-scattered electron mode (BSE). Magnifications: (a) x2500, (b) x1500, (c) x1600, (d) x3000, (e) x2500, (f) x1500. Location of EDS analyses marked with blue numbers and dots (see Tables S1 S2 for elemental and mineral composition);

Figure S8. Quartz grain size distribution of the faience body layer. (a–g) histograms of grain size distribution with lab numbers. Mass content = M_i / M, where M_i – mass of grains within the "i" interval, M – total mass of all grains. Compare with Figure 7 and Table S4;

Figure S9. Quartz grain shape distribution of the faience body layer. (a–g) histograms of the grain shape distribution with lab numbers. Grains with different shapes were distinguished based on the distribution of the form index K_f: isometric $a/b<1.5$, anisometric $1.5<a/b<10$ and elongated $a/b>10$ (a, b – extreme grain dimensions). Probability density of grains $Ph_i = N_i / Nxl$, where Ni – number of grains within the "i" interval, N – total number of grains, l – length of the interval;

Figure S10. Relationships between morphometric parameters of the quartz grains from the faience body layer. (a–d) correlations between selected parameters from Table 2. Dots marked with the same colour as the lines and lab numbers in Figure 7 and Tables 2, S4.

Table S1. Elemental composition of the accessory grains from the faience body and glaze layer. See Figures 4, S3-S7 for location of EDS analyses;

Table S2. Data base of SEM-EDS analyses of the accessory grains from the faience body and glaze layer;

Table S3. Microstructural and microtextural data of the quartz grains from the faience body layer (compare with images in Figures S1, 8, 9);

Table S4. Quartz grain size composition of the faience body layer. Lab numbers marked with the same colour as the lines in Figure 7 (note value of mass content for different size ranges and compare with the same data).
