# Supplementary material for: Source of Raw Materials and Its Processing for the Manufacturing of Ptolemaic Faience Bowls from Tell Atrib (Nile Delta, Egypt)
Source: Materials (Basel). 2022 Sep 8;15(18):6251. doi: 10.3390/ma15186251 (PMC9501641; doi:10.3390/ma15186251)
Supplement: Supplementary file 1 [file materials-15-06251-s001.zip › Supplementary materials_revised/Table S3.pdf]

| Lab no. | Size (µm) | Shape | Roundness | Sharpness | Cavities | Cracks |
|---|---|---|---|---|---|---|
| **NZA** | <50 +++<br>>50 + | EL +<br>AN +++<br>IS ++ | VA ++<br>A +++<br>S-A ++ | EG +<br>CO +<br>JG ++ | +<br>oval | +++ outline<br>+ across |
| **B37** | <50 +++<br>>50 + | EL +<br>AN +++<br>IS ++ | VA +++<br>A +<br>S-A – | EG +++<br>CO +++<br>JG + | ++<br>oval | + outline<br>++ across |
| **B9.1** | <50 + thicker<br>>50 +++<br>>100 + | EL +<br>AN +++<br>IS ++ | VA ++<br>A ++<br>S-A + | EG ++<br>CO ++<br>JG + | ++<br>oval | ++ outline<br>+ across |
| **B9.2** | <50/>50 | EL +<br>AN +++<br>IS ++ | VA ++<br>A +++<br>S-A + | EG +<br>CO ++<br>JG + | ++<br>oval<br>triangular | ++ outline<br>+ across |
| **MS** | <50 + thicker<br>>50 +++<br>>100 + | EL +<br>AN +++<br>IS ++ | VA +<br>A ++<br>S-A +++ | EG +<br>CO +<br>JG – | +<br>oval<br>triangular | – outline<br>+ across |
| **B100** | <50 +<br>>50 +++<br>>100 + | EL +<br>AN +++<br>IS ++ | VA +<br>A ++<br>S-A + | EG –<br>CO +<br>JG ++ | +<br>oval<br>triangular | +++ outline<br>+ across |
| **B81** | < 50/>50 | EL +<br>AN +++<br>IS ++ | VA ++<br>A +++<br>S-A + | EG +<br>CO ++<br>JG + | +<br>oval<br>triangular | ++ outline<br>+ across |

qualitative analysis: +++ high, ++ medium, + low, / similar, – no; EL – elongated, AN – anisometric, IS – isometric; VA – very angular, A – angular, S-A – sub-angular, EG – edges, CO – corners, JG – jagged
