# Supplementary material for: Source of Raw Materials and Its Processing for the Manufacturing of Ptolemaic Faience Bowls from Tell Atrib (Nile Delta, Egypt)
Source: Materials (Basel). 2022 Sep 8;15(18):6251. doi: 10.3390/ma15186251 (PMC9501641; doi:10.3390/ma15186251)
Supplement: Supplementary file 1 [file materials-15-06251-s001.zip › Supplementary materials_revised/Table S4.pdf]

Size class ø [mm]	Size range ø [mm]	Lab no.													
		NZA		B37		B9.1		B9.2		MS		B100		B81	
		Mass content [%]													
Sand 2.0 – 0.05	0.3 – 0.2	**27.4**	0.0	29.4	0.0	**65.5**	11.8	**56.9**	0.0	**72.6**	9.5	**79.1**	9.8	**56.6**	0.0
	0.2 – 0.1		4.8		3.6		26.9		27.0		25.6		36.5		22.4
	0.1 – 0.07		8.7		9.4		13.2		15.2		17.6		19.2		16.5
	0.07 – 0.05		13.9		16.4		13.7		14.7		19.9		13.7		17.8
Silt 0.05 – 0.002	0.05 – 0.04	**72.6**	13.2	70.5	9.9	**34.5**	8.8	**43.1**	9.0	**27.4**	9.2	**20.9**	6.6	**43.4**	10.7
	0.04 – 0.03		17.7		15.7		9.8		10.5		8.8		5.6		10.0
	0.03 – 0.02		19.7		17.9		9.1		12.1		6.0		4.9		10.8
	0.02 – 0.01		18.6		19.9		5.9		9.4		2.9		3.0		9.5
	0.01– 0.007		2.1		3.9		0.6		1.4		0.3		0.4		1.7
	0.007 – 0.005		0.8		1.9		0.2		0.5		0.1		0.2		0.6
	0.005 – 0.002		0.5		1.3		0.1		0.3		0.1		0.2		0.2
Clay <0.002	0.002 – 0.001	**0.0**	0.0	**0.1**	0.1	**0.0**	0.0	**0.0**	0.0	**0.0**	0.0	**0.0**	0.0	**0.0**	0.0
	0.001 – 0.000		0.0		0.0		0.0		0.0		0.0		0.0		0.0
