# Supplementary figures and images for: Source of Raw Materials and Its Processing for the Manufacturing of Ptolemaic Faience Bowls from Tell Atrib (Nile Delta, Egypt)
Source: Materials (Basel). 2022 Sep 8;15(18):6251. doi: 10.3390/ma15186251 (PMC9501641; doi:10.3390/ma15186251)

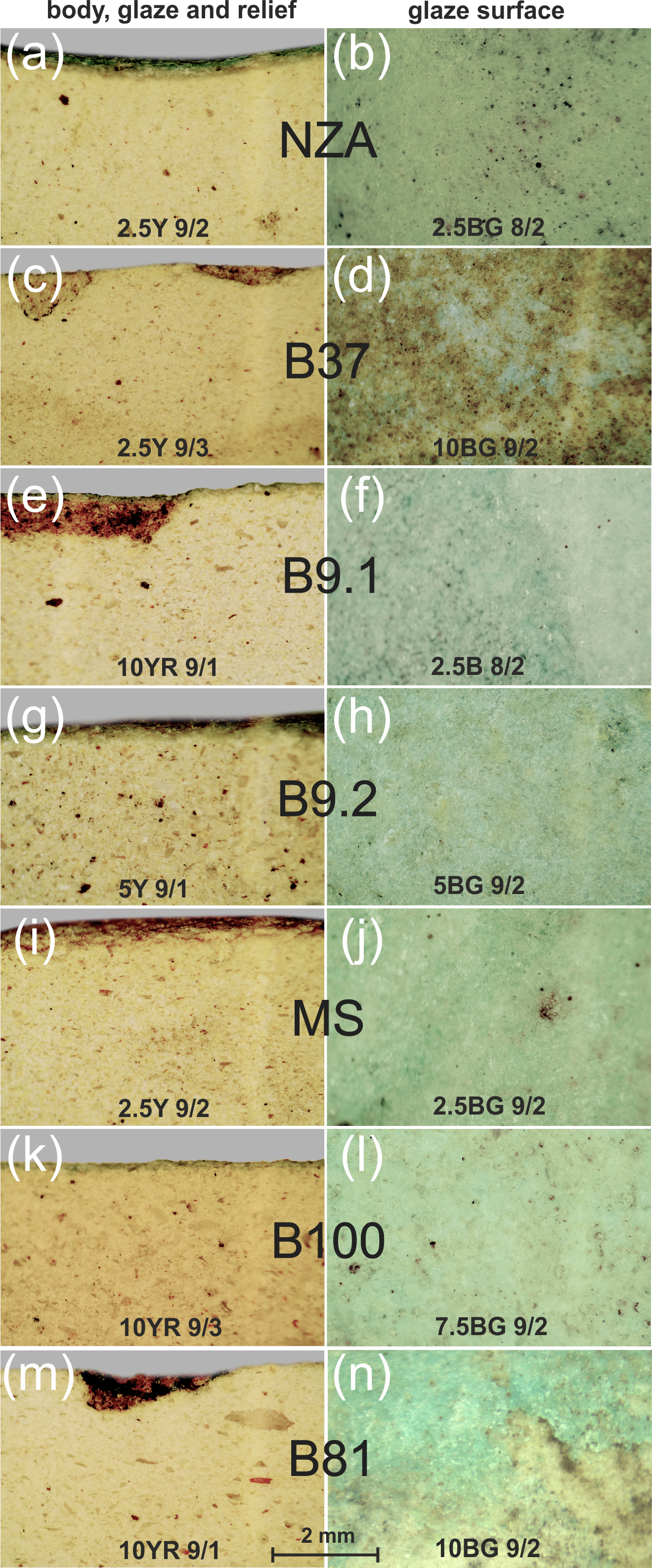

Supplement: Supplementary file 1 [file materials-15-06251-s001.zip › Supplementary materials_revised/Fig. S1.png]

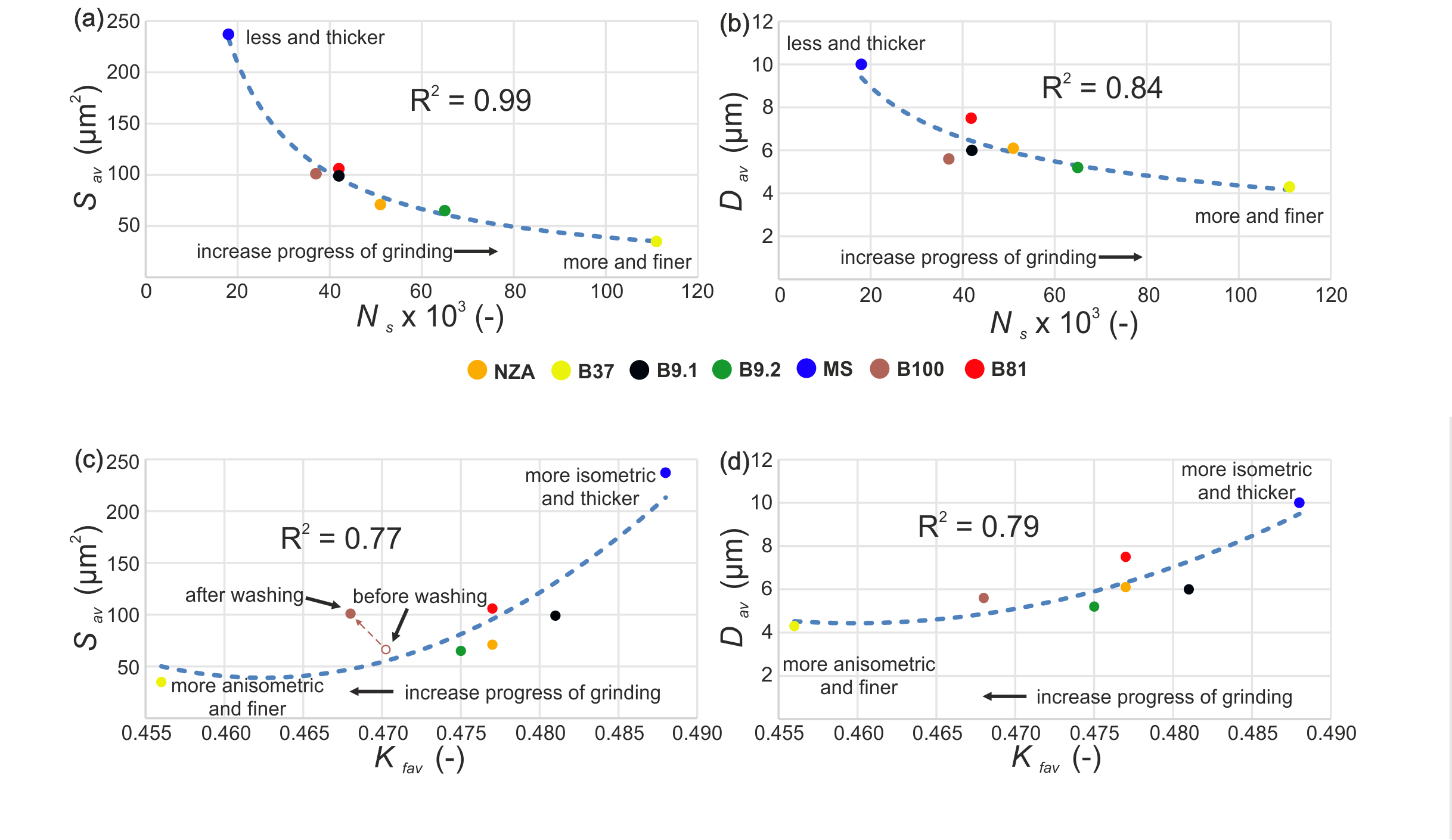

Supplement: Supplementary file 1 [file materials-15-06251-s001.zip › Supplementary materials_revised/Fig. S10.png]

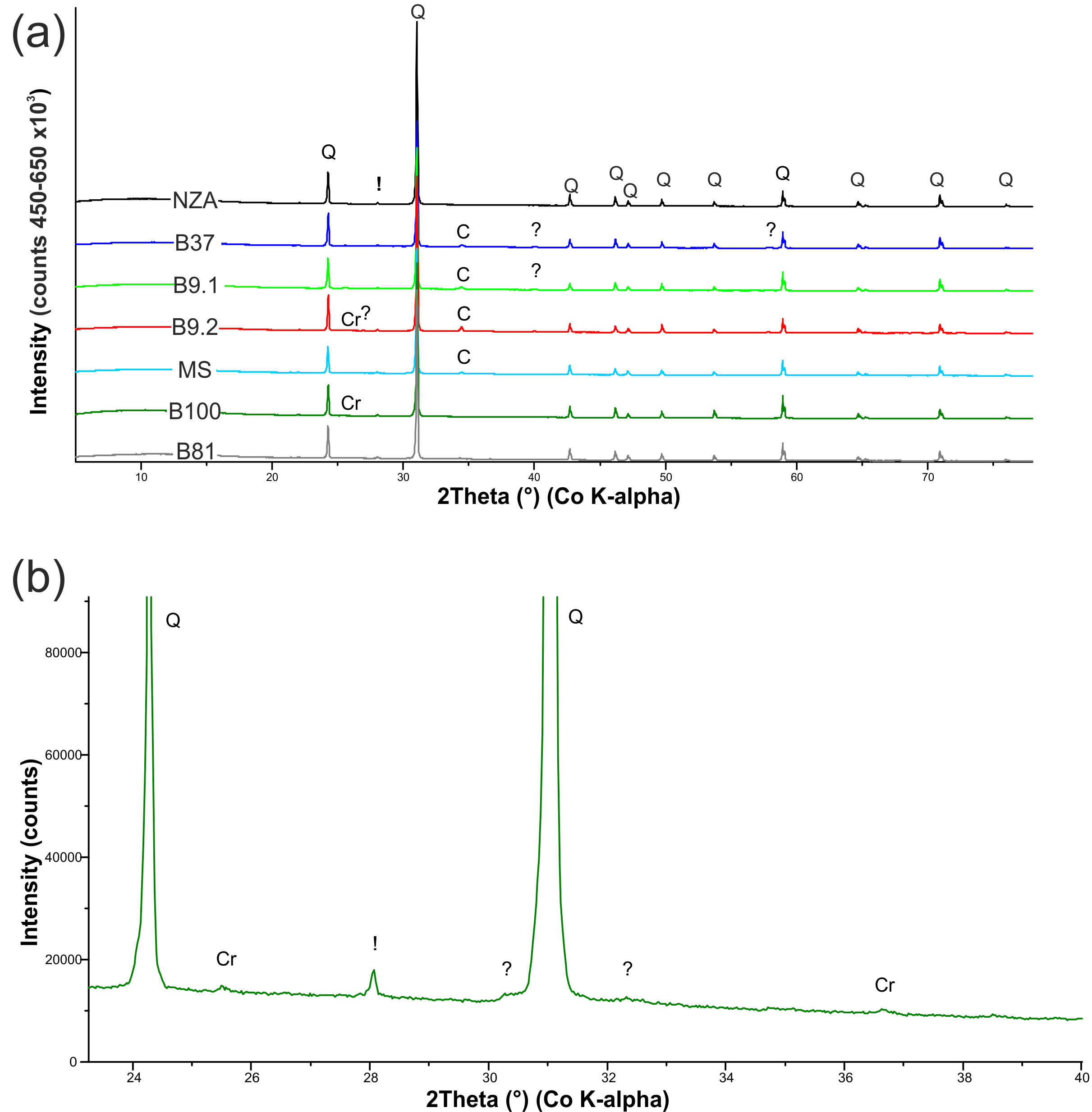

Supplement: Supplementary file 1 [file materials-15-06251-s001.zip › Supplementary materials_revised/Fig. S2.png]

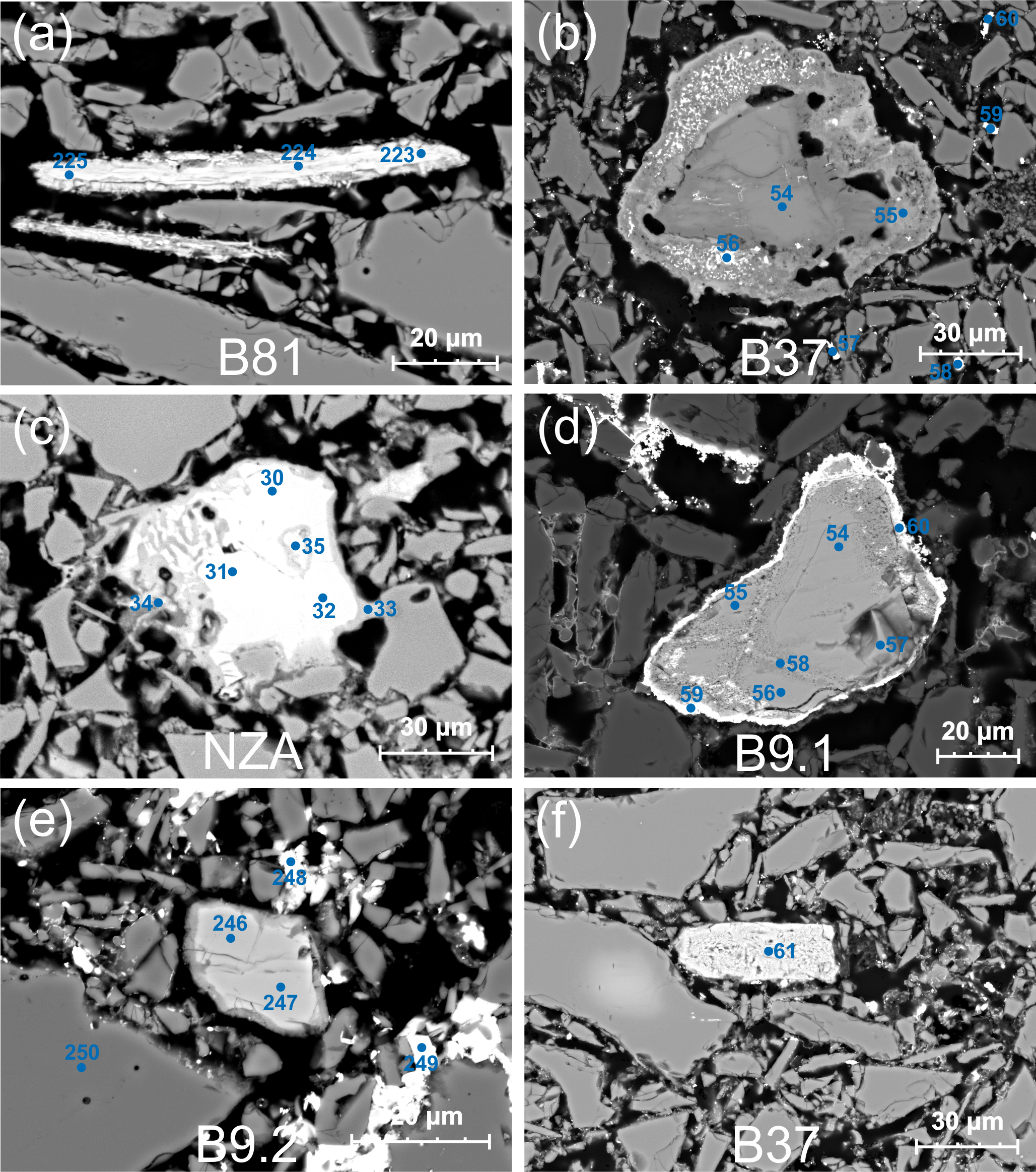

Supplement: Supplementary file 1 [file materials-15-06251-s001.zip › Supplementary materials_revised/Fig. S3.png]

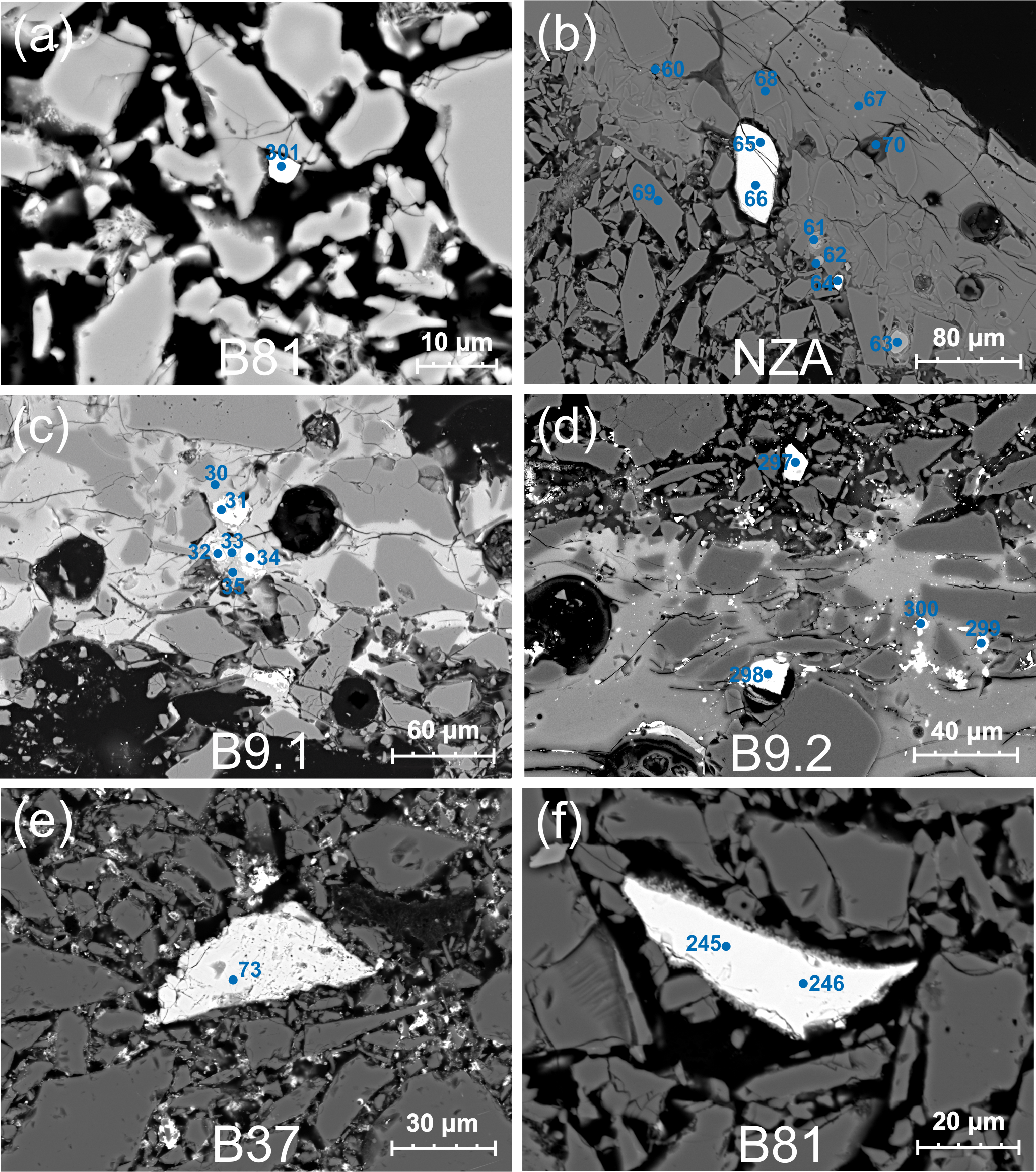

Supplement: Supplementary file 1 [file materials-15-06251-s001.zip › Supplementary materials_revised/Fig. S4.png]

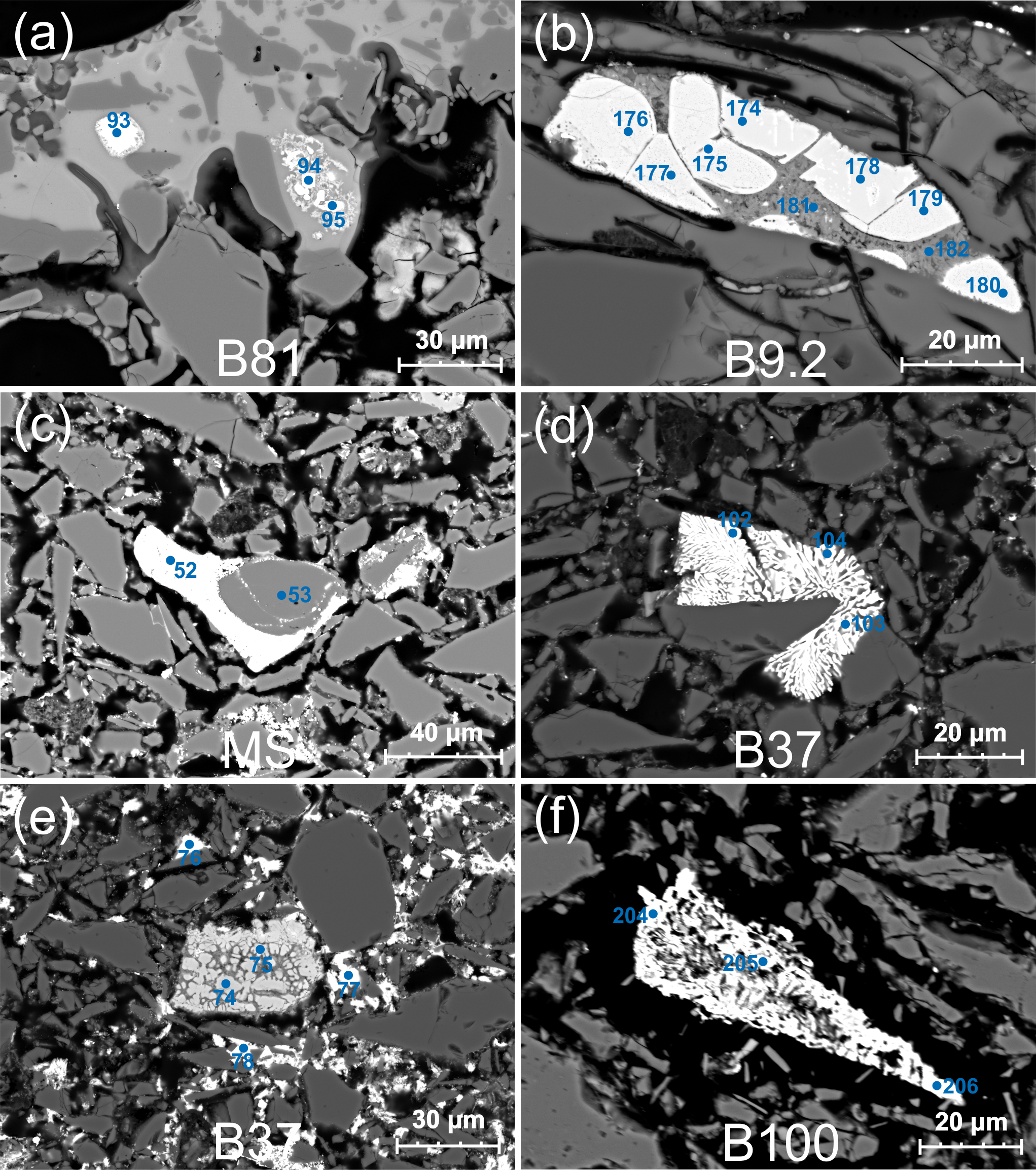

Supplement: Supplementary file 1 [file materials-15-06251-s001.zip › Supplementary materials_revised/Fig. S5.png]

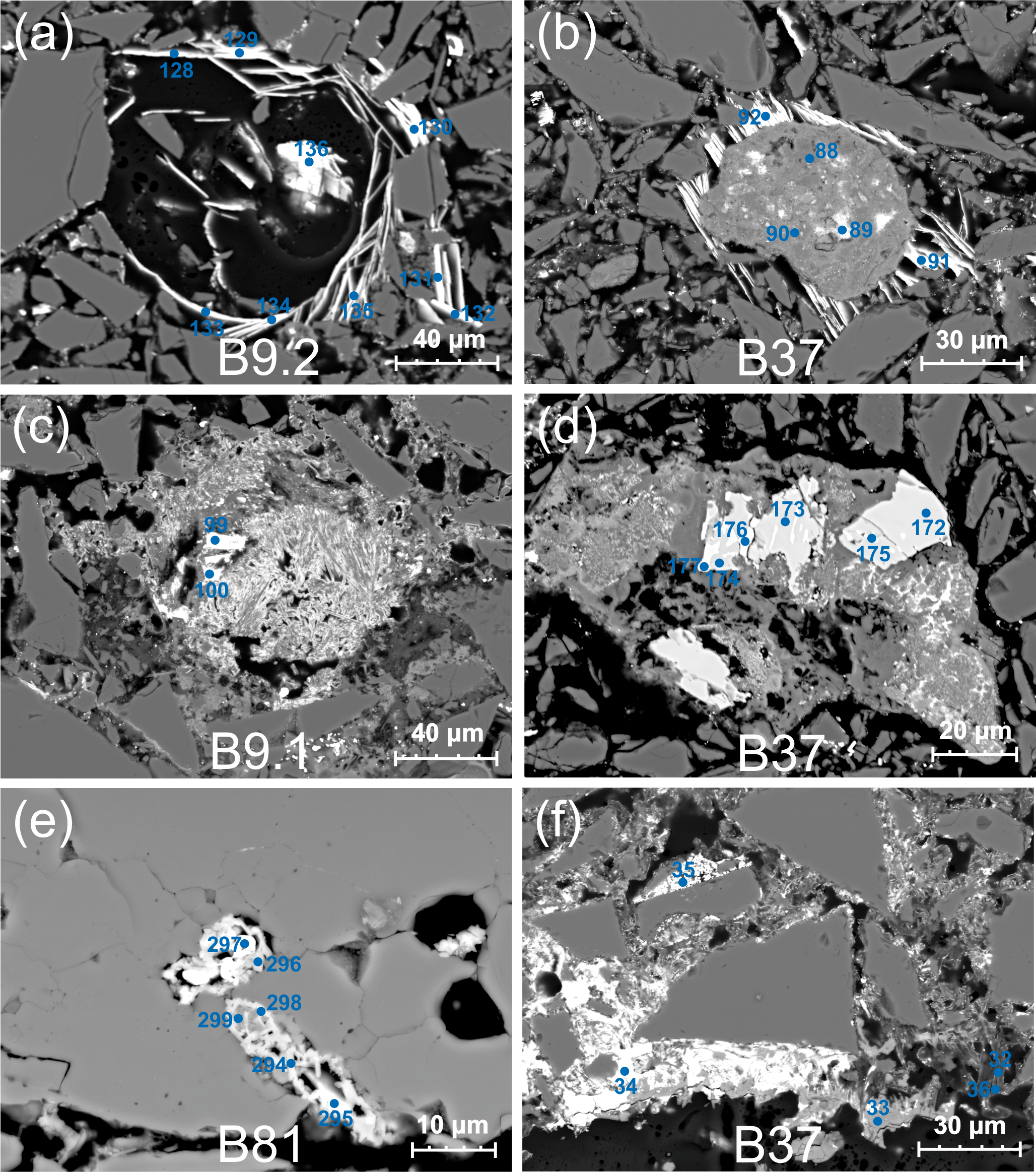

Supplement: Supplementary file 1 [file materials-15-06251-s001.zip › Supplementary materials_revised/Fig. S6.png]

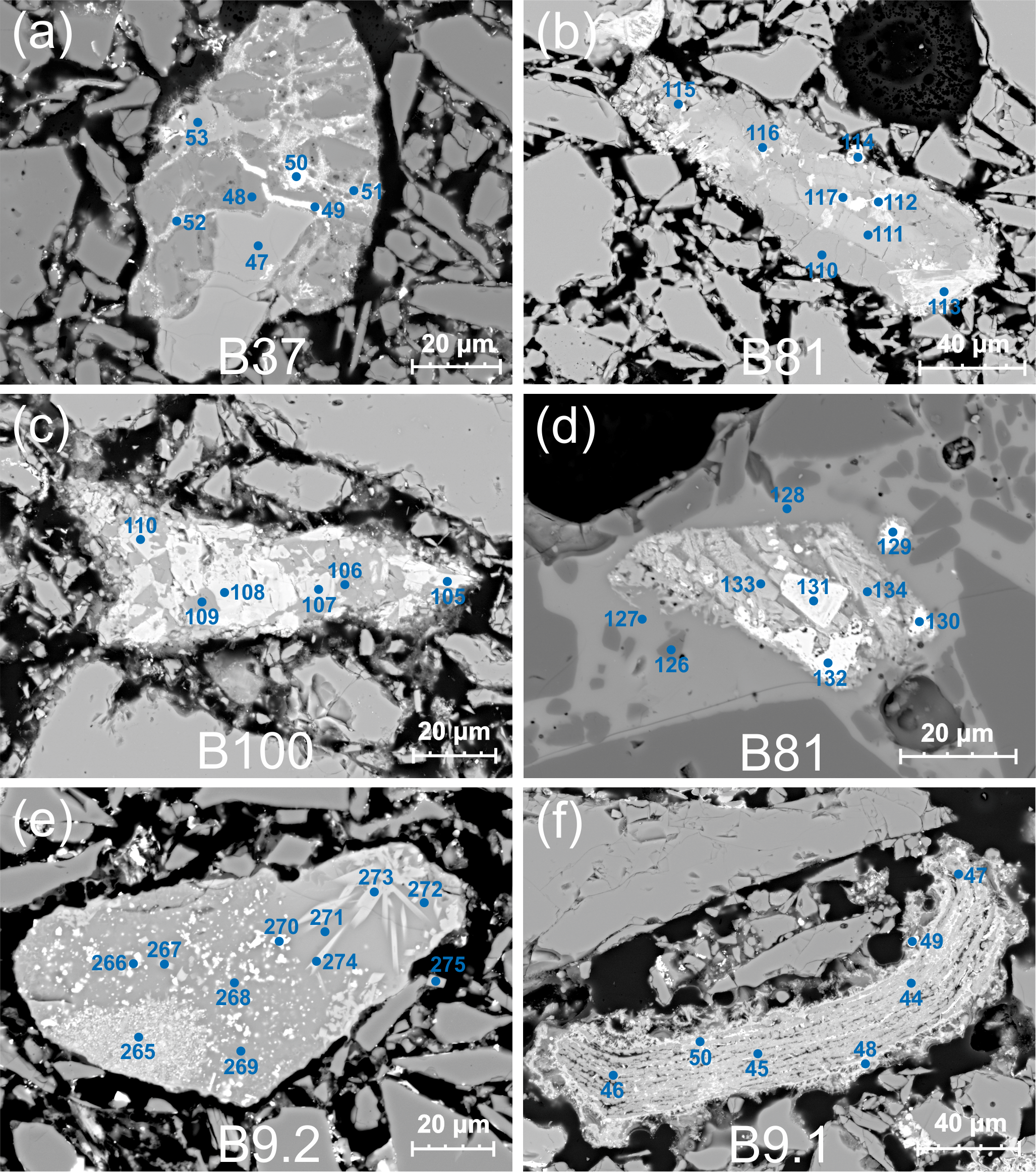

Supplement: Supplementary file 1 [file materials-15-06251-s001.zip › Supplementary materials_revised/Fig. S7.png]

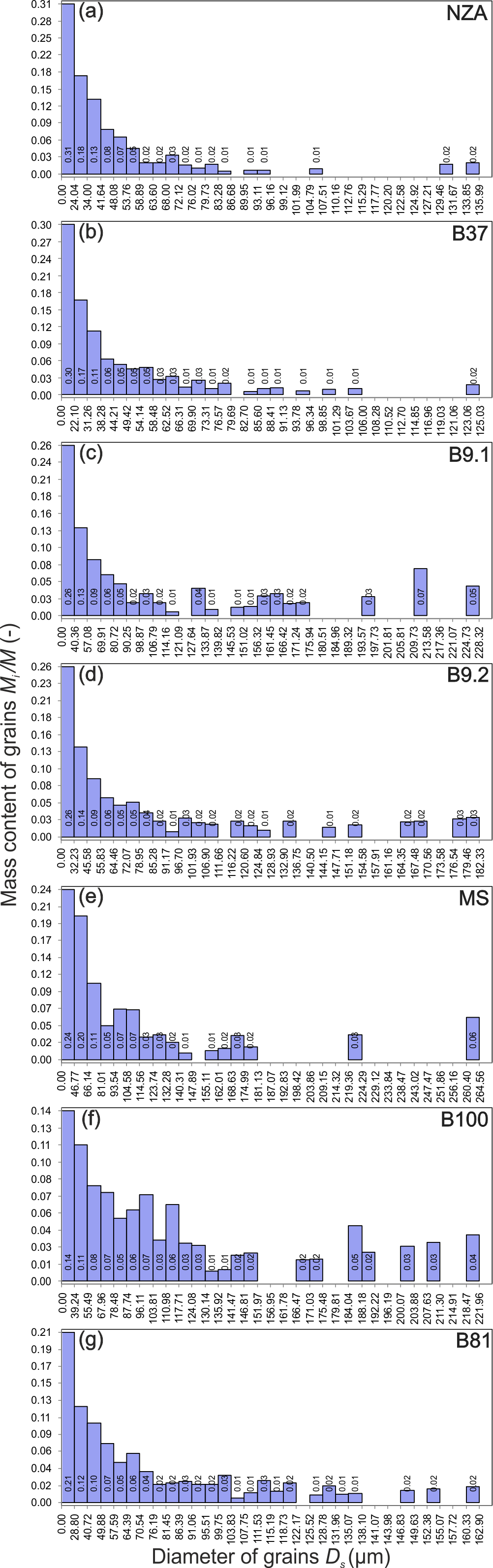

Supplement: Supplementary file 1 [file materials-15-06251-s001.zip › Supplementary materials_revised/Fig. S8.png]

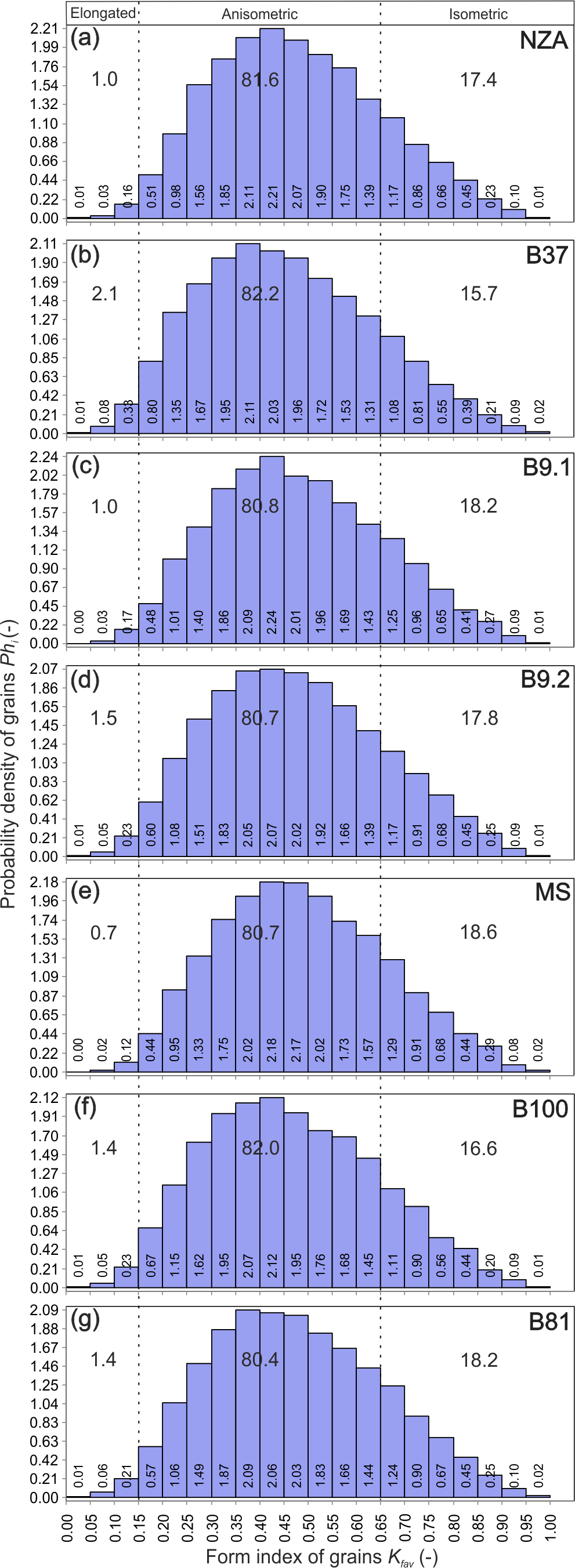

Supplement: Supplementary file 1 [file materials-15-06251-s001.zip › Supplementary materials_revised/Fig. S9.png]
